# Network-based Survival Analysis Reveals Subnetwork Signatures for Predicting Outcomes of Ovarian Cancer Treatment

**DOI:** 10.1371/journal.pcbi.1002975

**Published:** 2013-03-21

**Authors:** Wei Zhang, Takayo Ota, Viji Shridhar, Jeremy Chien, Baolin Wu, Rui Kuang

**Affiliations:** 1 Department of Computer Science and Engineering, University of Minnesota Twin Cities, Minneapolis, Minnesota, United States of America; 2 Department of Laboratory Medicine and Experimental Pathology, Mayo Clinic College of Medicine, Rochester, Minnesota, United States of America; 3 Division of Biostatistics, School of Public Health, University of Minnesota Twin Cities, Minneapolis, Minnesota, United States of America; University of Tokyo, Japan

## Abstract

Cox regression is commonly used to predict the outcome by the time to an event of interest and in addition, identify relevant features for survival analysis in cancer genomics. Due to the high-dimensionality of high-throughput genomic data, existing Cox models trained on any particular dataset usually generalize poorly to other independent datasets. In this paper, we propose a network-based Cox regression model called Net-Cox and applied Net-Cox for a large-scale survival analysis across multiple ovarian cancer datasets. Net-Cox integrates gene network information into the Cox's proportional hazard model to explore the co-expression or functional relation among high-dimensional gene expression features in the gene network. Net-Cox was applied to analyze three independent gene expression datasets including the TCGA ovarian cancer dataset and two other public ovarian cancer datasets. Net-Cox with the network information from gene co-expression or functional relations identified highly consistent signature genes across the three datasets, and because of the better generalization across the datasets, Net-Cox also consistently improved the accuracy of survival prediction over the Cox models regularized by 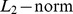 or 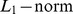. This study focused on analyzing the death and recurrence outcomes in the treatment of ovarian carcinoma to identify signature genes that can more reliably predict the events. The signature genes comprise dense protein-protein interaction subnetworks, enriched by extracellular matrix receptors and modulators or by nuclear signaling components downstream of extracellular signal-regulated kinases. In the laboratory validation of the signature genes, a tumor array experiment by protein staining on an independent patient cohort from Mayo Clinic showed that the protein expression of the signature gene FBN1 is a biomarker significantly associated with the early recurrence after 12 months of the treatment in the ovarian cancer patients who are initially sensitive to chemotherapy. Net-Cox toolbox is available at http://compbio.cs.umn.edu/Net-Cox/.

## Author Summary

Network-based computational models are attracting increasing attention in studying cancer genomics because molecular networks provide valuable information on the functional organizations of molecules in cells. Survival analysis mostly with the Cox proportional hazard model is widely used to predict or correlate gene expressions with time to an event of interest (outcome) in cancer genomics. Surprisingly, network-based survival analysis has not received enough attention. In this paper, we studied resistance to chemotherapy in ovarian cancer with a network-based Cox model, called Net-Cox. The experiments confirm that networks representing gene co-expression or functional relations can be used to improve the accuracy and the robustness of survival prediction of outcome in ovarian cancer treatment. The study also revealed subnetwork signatures that are enriched by extracellular matrix receptors and modulators and the downstream nuclear signaling components of extracellular signal-regulators, respectively. In particular, FBN1, which was detected as a signature gene of high confidence by Net-Cox with network information, was validated as a biomarker for predicting early recurrence in platinum-sensitive ovarian cancer patients in laboratory.

## Introduction

Survival analysis is routinely applied to analyzing microarray gene expressions to assess cancer outcomes by the time to an event of interest [1]–[3]. By uncovering the relationship between gene expression profiles and time to an event such as recurrence or death, a good survival model is expected to achieve more accurate prognoses or diagnoses, and in addition, to identify genes that are relevant to or predictive of the events [4], [5]. The Cox proportional hazard model [6] is widely used in survival analysis because of its intuitive likelihood modeling with both uncensored patient samples and censored patient samples who are event-free by the last follow-up. Due to the high dimensionality of typical microarray gene expressions, the Cox regression model is usually regularized with penalties such as 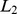 penalty in ridge regression [7]–[10], 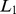 Lasso regularization [11]–[16] and 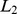 regularization in Hilbert space [17]. While those penalties were designed as a statistical or algorithmic treatment for the high-dimensionality problem, these Cox models are still prone to noise and overfitting to the low sample size. An important prior information that has been largely ignored in survival analysis is the modular relations among gene expressions. Groups of genes are co-expressed under certain conditions or their protein products interact with each other to carry out a biological function. It has been shown that protein-protein interaction network or co-expressions can provide useful prior knowledge to remove statistical randomness and confounding factors from high-dimensional data for several classification and regression models [18]–[21]. The major advantage of these network-based models is the better generalization across independent studies since the network information is consistent with the conserved patterns in the gene expression data. For example, previous studies in [18], [20] discovered that more consistent signature genes of breast cancer metastasis can be identified from independent gene expression datasets by network-based classification models. The observations also motivated several graph algorithms for detecting cancer causal genes in protein-protein interaction network [22], [23].

**Figure 1:**
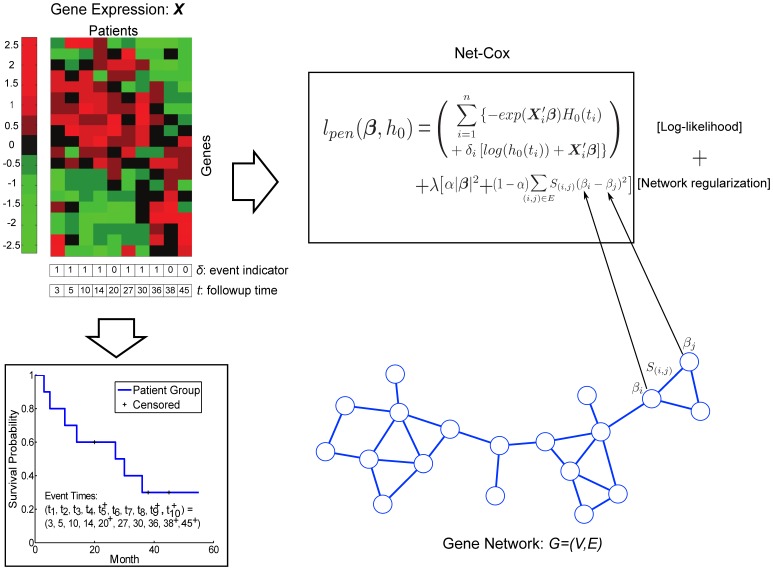
Overview of Net-Cox. The patient gene expression data 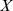 and the survival information specified by followup times 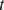 and event indicators 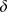 are illustrated on the left. The cost function of Net-Cox given in the box combines the total likelihood of Cox regression with a network regularization. The gene network shown is used as a constraint to encourage smoothness among correlated genes, i.e. the coefficients of the genes connected with edges of large weights are similarly weighted.

In this article, we propose a network-based Cox proportional hazard model called Net-Cox to explore the co-expression or functional relation among gene expression features for survival analysis. The relation between gene expressions are modeled by a gene relation network constructed by co-expression analysis or prior knowledge of gene functional relations. In the Net-Cox model, a graph Laplacian constraint is introduced as a smoothness requirement on the gene features linked in the gene relation network. Figure 1 illustrates the general framework of Net-Cox for utilizing gene network information in survival analysis. In the framework, the cost function of Net-Cox, shown in the box, combines the total likelihood of Cox regression with a network regularization. The total log-likelihood is a function of the linear regression coefficients 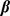 and the base hazard 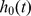 on each followup time 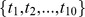, represented by the likelihood ratios with the patient gene expression data and the survival information specified by followup times and event indicators. The gene network is either constructed with gene co-expression information or a given gene functional linkage network. The gene network is modeled as a constraint to encourage smoothness among correlated genes, for example gene 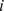 and 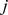 in the network, such that the coefficients of the genes connected with edges of large weights are similarly weighted. The cost function of Net-Cox can be solved by alternating optimization of 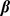 and 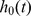 by iterations. An algorithm that solves the Net-Cox model in its dual representation is also introduced to improve the efficiency. The complete model is explained in detail in Section **Materials and Methods**.

In this study, we applied Net-Cox to identify gene expression signatures associated with the outcomes of death and recurrence in the treatment of ovarian carcinoma. Ovarian cancer is the fifth-leading cause of cancer death in US women [3]. Identifying molecular signatures for patient survival or tumor recurrence can potentially improve diagnosis and prognosis of ovarian cancer. Net-Cox was applied on three large-scale ovarian cancer gene expression datasets [3], [24], [25] to predict survivals or recurrences and to identify the genes that may be relevant to the events. Our study is fundamentally different from previous survival analysis on ovarian cancer [3], [24]–[26], which are based on univariate Cox regression. For example, in [3], gene expression profiles from 215 stage II–IV ovarian tumors from TCGA were used to identify a prognostic gene signature (univariate Cox 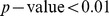) for overall survival, including 108 genes correlated with poor (worse) prognosis and 85 genes correlated with good (better) prognosis. In [24], a Cox score is defined to measure the correlation between gene expression and survival. The genes with a Cox score that exceeds an empirically optimized threshold in leave-one-out cross-validation were reported as signature genes. Similarly, in [25] and [26], a univariate Cox model was applied to identify association between gene expressions and survival (univariate Cox 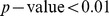). Our study is based on gene networks enriched by co-expression and functional information and thus identifies subnetwork signatures for predicting survival or recurrence in ovarian cancer treatment.

## Results

In the experiments, Net-Cox was applied to analyze three ovarian cancer gene expression datasets listed in Table 1. Net-Cox (equation (9)) was compared with 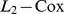 (equation (6)) and 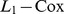 (equation (7)) with performance evaluation in survival prediction and gene signature identification for the analysis of patient survival and tumor recurrence. First, for evaluation with a better focus on cancer-relevant genes, the expressions of a list of 2647 genes that are previously known to be related to cancer (Sloan-Kettering cancer genes) are used. On the data of these 2647 genes, Net-Cox, 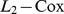 and 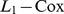 were evaluated by consistency of signature gene selection across the three datasets, accuracy of survival prediction and assessment of statistical significance. Next, more comprehensive experiments on all 7562 mappable genes were conducted to identify novel signature genes associated with ovarian cancer. Finally, we further analyzed and validated ovarian cancer signatures by an additional tumor array experiment and literature survey. In all the experiments, gene co-expression networks and a gene functional linkage network were used to derive the network constraints for Net-Cox. The details of data preparation and the algorithms are described in Section **Materials and Methods**.

**Table 1:** Patient samples in the ovarian cancer datasets.

	Dataset (GEO ID)	TCGA (N/A)	Tothill (GSE9899)	Bonome (GSE26712)
**Death**	**# of Censored**	227	160	24
	**# of Uncensored**	277	111	129
**Recurrence**	**# of Censored**	241	86	N/A
	**# of Uncensored**	263	185	N/A

The number of patients categorized by censoring and uncensoring for the death and recurrent events is reported in each dataset. Note that the Bonome dataset does not provide information on recurrence.

### Net-Cox identifies consistent signature genes across independent datasets

To evaluate the generalization of the models, we first measured the consistency among the signature genes selected from the three independent datasets by each method. Specifically, we report the percentage of common genes in the three rank lists identified by a method. This measurement assumes that even under the presence of biological variability in gene expressions and patient heterogeneities in each dataset, genes that are selected in multiple datasets are more likely to be true signature genes. Thus, higher consistency across the datasets might indicate higher quality in gene selection.

**Figure 2:**
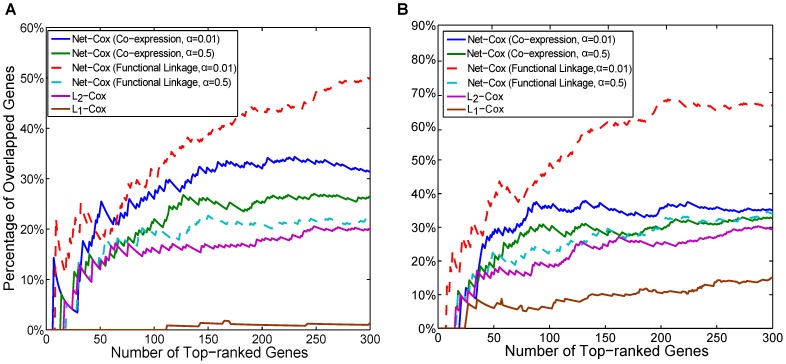
Consistency of signature genes (Sloan-Kettering cancer genes). The x-axis is the number of selected signature genes ranked by each method. The y-axis is the percentage of the overlapped genes between the selected genes across the ovarian cancer datasets. The plots show the results for the death outcome (A) and the tumor recurrence outcome (B).

In Figure 2, we plot the number of common genes among the first 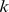 (up to 300) genes in the gene ranking lists from all of the three datasets for the death event and two datasets (TCGA and Tothill) for the recurrence event. For the parameter setting of Net-Cox, we fixed 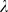 to be the optimal parameter in the five-fold cross-validation (see Section **Materials and Methods** and report the results with 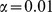 and 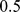. Since the ranking lists of Net-Cox with 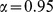 are nearly identical to those of 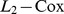, they are not reported for better clarity in the figure. The first observation is that the gene rankings by Net-Cox are more consistent than those by 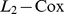 and 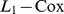 at all the cutoffs. Moreover, Net-Cox with 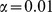 identified more common signature genes than Net-Cox with 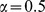. For example, for the tumor recurrence outcome, Net-Cox (Co-expression) with 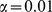 and 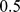 identified 36 and 29 common genes among the first 100 genes in the gene ranking lists, Net-Cox (Functional linkage) with 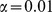 and 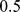 identified 49 and 23 common genes, and 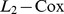 and 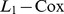 only identified 19 and 6 common genes, respectively. In general, variable selection by 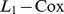 is not stable from high-dimensional gene expression data, and thus, the overlaps in the gene lists by 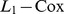 are significantly lower than the other methods. It is also interesting to see the gradient of the overlap ratio from 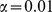 to 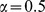, and then to 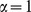 (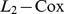), which indicates that, when a gene network plays more an important role in gene selection, the gene rankings tend to be more consistent. This observation is consistent with previous studies with protein-protein interaction network or gene co-expression network [18], [20], [21]. Note that since the overlaps are across three datasets for the death event and across two datasets for the recurrence event, the overlaps for the death event is expected to be lower than those for the recurrence event. Another important difference is that the same functional linkage network is always used while the co-expression network is dataset-specific. Thus, it is also expected that the overlaps by Net-Cox with the functional linkage network is higher than those by Net-Cox with the co-expression network. Together, the results demonstrate that Net-Cox effectively utilized the network information to improve gene selection and accordingly, the generalization of the model to independent data.

### Net-Cox improves survival prediction across independent datasets

**Figure 3:**
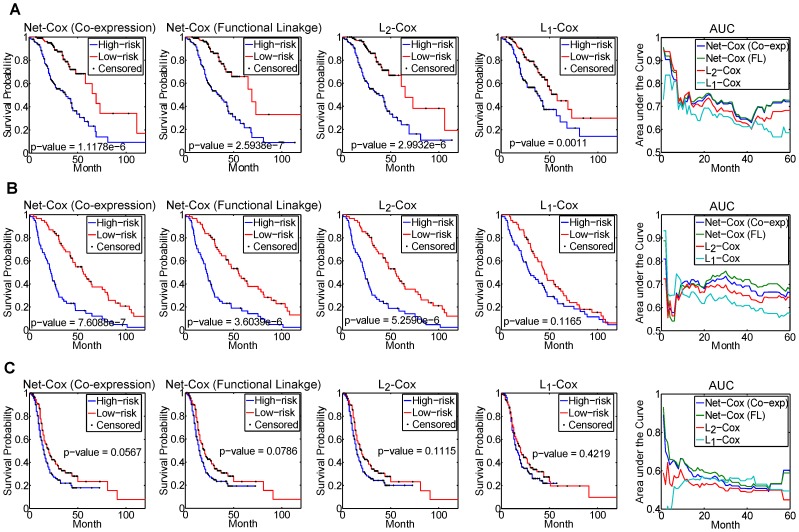
Cross-dataset survival prediction (Sloan Kettering cancer genes). The first four columns of plots show the Kaplan-Meier survival curves for the two risk groups defined by Net-Cox (co-expression network), Net-Cox (functional linkage network), 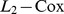 and 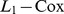. The fifth column of plots compare the time-dependent area under the ROC curves based on the estimated risk scores (PIs). The plots show the results for the death outcome by training with TCGA dataset and test on Tothill Dataset (A), the death outcome by training with TCGA dataset and test on Bonome Dataset (B), the tumor recurrence outcome by training with TCGA dataset and test on Tothill Dataset (C).

Five-fold cross-validation was first conducted for parameter tuning for Net-Cox, 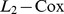 and 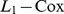 on each dataset. The optimal parameters of Net-Cox are reported in Table S1. To test how well the models generalize across the datasets, we trained Net-Cox model, 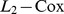 model, and 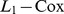 model with the TCGA dataset, and then predicted the survival of the patients in the other two datasets with the TCGA-trained models. In training, we used the optimal 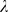 and 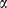 from the five-fold cross-validation to train the models with the whole TCGA dataset. The results are given in Table 2. In all the cases, Net-Cox obtained more significant 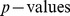 in the log-rank test than 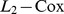 and 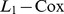. To further compare the results, we show the Kaplan-Meier survival curves and the ROC curves in Figure 3. The first four columns of plots in the figure show the Kaplan-Meier survival curves for the two risk groups defined by Net-Cox with co-expression network and functional linkage network, 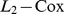, and 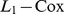. The fifth column of plots compare the time-dependent area under the ROC curves based on the estimated risk scores (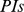). In Figure 3, in many regions, Net-Cox achieved large improvement over both 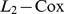 and 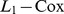 while the improvement is less obvious in several other regions. Overall, Net-Cox achieved better or similar AUCs in all the time points in the three plots. To evaluate the statistical significance of the differences between the time-dependent AUCs generated by Net-Cox and the other two methods, in Table S2 we report 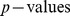 at each event time with the null hypothesis that the two time-dependent AUCs estimated by two models are equal. At many points of the event time, the time-dependent AUCs generated from Net-Cox are significant higher.

**Table 2:** Log-rank test 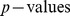 in cross-dataset evaluation (Sloan-Kettering cancer genes).

	Test Dataset	Net-Cox (Co-exp)	Net-Cox (FL)	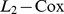	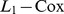
**Death**	**Tothill**	1.1178E-06	2.5938E-07	2.9932E-06	0.0011
	**Bonome**	7.6088E-07	3.6039E-06	5.2590E-06	0.1165
**Recurrence**	**Tothill**	0.0567	0.0786	0.1115	0.4219

The survival prediction performance on Tothill and Bonome datasets using the Cox models trained with TCGA dataset are reported.

The cross-validation log-partial likelihood (CVPLs) for the combinations of 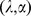 in the five-fold cross-validation are also reported in Table S3. In all the cases, the optimal CVPLs of Net-Cox are higher than those of 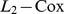. 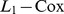 was fine-tuned with 1000 choices of parameters with a very small bin size. In one of the cases (TCGA: Recurrence), the optimal CVPL of 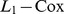 is higher but in the other cases, the optimal CVPLs of Net-Cox are higher. Interestingly, the optimal 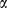 is often 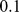 or 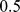, indicating the optimal CVPL is a balance of the information from gene expressions and the network. The observations prove that the network information is useful for improving survival analysis. The left column of Figure S1 shows the average time-dependent area under the ROC curves based on the estimated risk scores (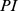) of the patients in the fifth fold of the five repeats, and Table S4A and S4B show log-rank 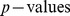 of the fifth fold of the five repeats. Net-Cox achieved the best overall survival prediction although the results are less obvious than those of the cross-dataset analysis.

### Statistical assessment

**Figure 4:**
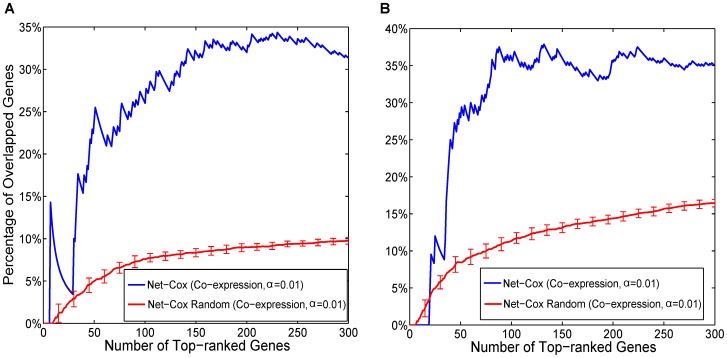
Consistency of signature genes on randomized co-expression networks. The x-axis is the number of selected signature genes ranked by each method. The y-axis is the percentage of the overlapped genes between the selected genes across the ovarian cancer datasets. The red curve reports the mean and the standard deviation of the percentages averaged over the experiments of 50 randomized networks. The plots show the results for the death outcome (A) and the tumor recurrence outcome (B).

**Figure 5:**
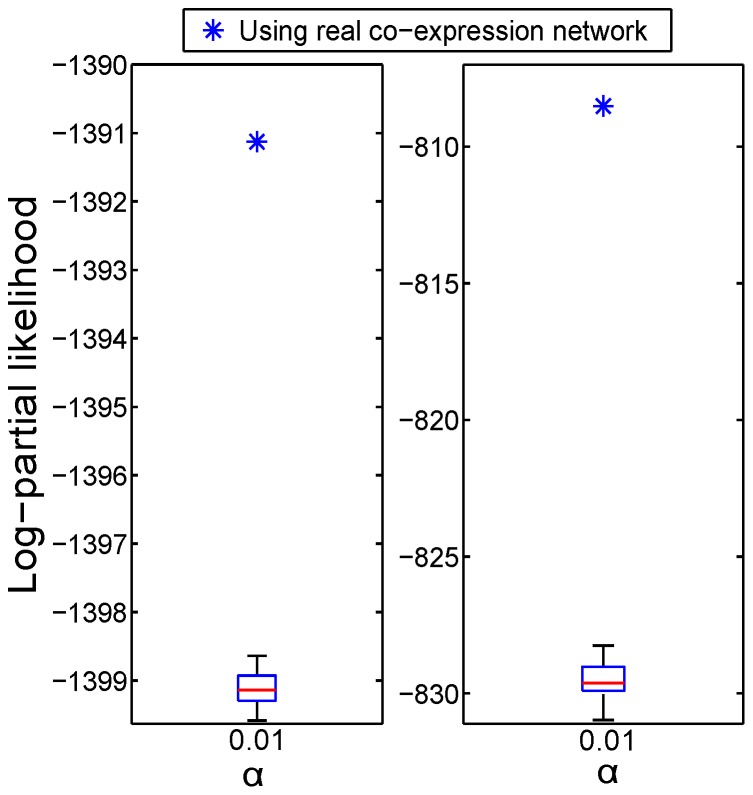
Statistical analysis of log-partial likelihood. The optimal 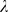 was fixed and 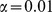 is set to allow better evaluation of the network information. The log-partial likelihood computed by Net-Cox on the real co-expression network and on the randomized co-expression network are reported against tumor recurrence in the TCGA and Tothill datasets. The stars represent the results with the real co-expression networks, and the boxplots represent the results with the randomized networks.

To understand the role of the gene network on the consistency in gene selection and the contribution to the log-partial likelihood, we tested Net-Cox with randomized co-expression networks. In each randomization, the weighted edges between genes were shuffled. We report the mean and the standard deviation of the percentage of overlapping genes of 50 randomizations in Figure 4. Compared with the consistency plots with the true networks, the overlaps by Net-Cox on the randomized networks are much lower. We also report the boxplot of the log-partial likelihood in the same 50 randomized co-expression network with 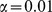 in Figure 5. Compare with the log-partial likelihood with the real co-expression network, the range of the likelihood generated with the randomized networks is again lower by a large margin, which provides clear evidence that the co-expression network is informative for survival analysis.

**Figure 6:**
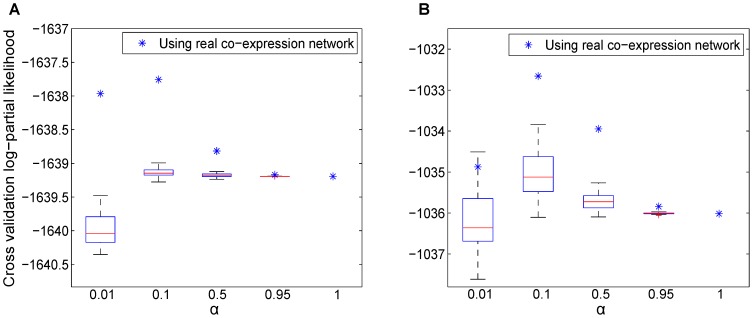
Statistical analysis of cross-validation log-partial likelihood (CVPL). The optimal 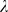 was fixed and 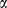 is varied from 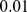 to 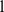. The CVPL of five-fold cross-validation on the real co-expression network and on the randomized co-expression network are reported against tumor recurrence in TCGA dataset (A) and Tothill dataset (B). The stars represent the results with the real co-expression networks, and the boxplots represent the results with the randomized networks.

To further understand the role of the network information in cross-validation, we fixed the optimal parameter 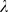 and conducted the same five-fold cross-validation with randomized co-expression networks to compute the CVPL with different 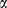 in {0.01, 0.1, 0.5. 0.95}. We repeated the process on 20 random networks for each 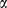. The boxplots of CVPLs with different 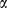
s are shown in Figure 6. In all measures, the CVPL with the true gene network is well above the mean of the 20 random cases. Another important observation is that, in both plots, when the randomized network information is more trusted with a smaller 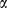, the variance of the CVPLs is also getting larger; and the case with 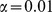 gives the worst CVPL mean and the largest variance. The result indicates that the randomized networks did not provide any valuable information in survival prediction. In contrast, with the true gene network, CVPLs generated from 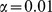 and 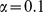 are much higher than the ones from 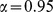 and 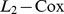 (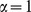). Again, these results convincingly support the importance of using the network information in survival prediction.

### Evaluation by whole gene expression data

Besides the 2647 Sloan-Kettering genes, all the 7562 mappable genes were also tested to evaluate Net-Cox, 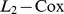 and 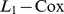 by consistency of signature gene selection across the three datasets and accuracy of survival prediction in similar experiments. For the signature gene consistency, Figure S2 reports the percentage of common genes identified by each method in the ranking lists from the datasets. For the cross-dataset validation, Table S5 shows the log-rank test 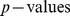 by training the TCGA datasets and test on the other two datasets, and Figure S3 shows the Kaplan-Meier survival curves for the two risk groups defined by Net-Cox, 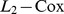 and 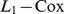 and compares the time-dependent area under the ROC curves. For the five-fold cross-validation, the right column of Figure S1 shows the average time-dependent area under the ROC curves based on the estimated risk scores (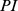) of the patients in the fifth fold of the five repeats, and Table S4C and S4D report log-rank test 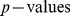 of the fifth fold of the five repeats. Overall, similar observations are made in experimenting with all the genes, though the improvements are less significant compared with the results by experimenting with the Sloan-Kettering cancer genes. One possible explanation is that, since the genes in the Sloan-Kettering gene list are more cancer relevant, the gene expressions may be more readily integrated with the network information.

### Signature genes are ECM components or modulators

To analyze the signature genes identified by Net-Cox and 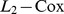, we created consensus rankings across the three datasets by re-ranking the genes with the lowest rank by Net-Cox and 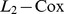 in the three datasets. Specifically, for each gene, a new ranking score is assigned as the lowest of its ranks in the three datasets, and then, all the genes were re-ranked by the new ranking score. The top-15 genes selected by Net-Cox and 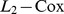 in the consensus rankings are shown in Table 3. For the death outcome, nine signature genes, FBN1, VCAN, SPARC, ADIPOQ, CNN1, DCN, LOX, EDNRA, LPL, known to be related to ovarian cancer [27]–[35] are only discovered by Net-Cox. Among the ten common genes highly ranked by both Net-Cox and 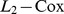, three are collagen genes, and MFAP5, TIMP3, THBS2, and CXCL12 are previously known to be relevant to ovarian cancer [36]–[39]. For the recurrence outcome, there are eleven common signature genes detected by both Net-Cox and 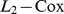. Net-Cox identified six additional ovarian cancer related signature genes [27]–[29], [40]–[42].

**Table 3:** Top-15 signature genes.

Death			Recurrence		
Net-Cox (Co-exp)	Net-Cox (FL)	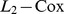	Net-Cox (Co-exp)	Net-Cox (FL)	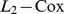
FBN1	COL11A1	COL11A1	COL5A2	COL11A1	COL11A1
COL5A2	MFAP4	FABP4	COL1A1	COL10A1	NLRP2
VCAN	TIMP3	MFAP4	COL5A1	CRYAB	CRYAB
SPARC	MFAP5	COMP	THBS2	NPY	PTX3
AEBP1	COL5A2	BCHE	FAP	IGF1	COL10A1
AOC3	THBS2	FAP	COL3A1	COMP	CXCL12
COL3A1	FAP	COL5A2	COL11A1	KLK5	THBS2
THBS2	CXCL12	MFAP5	FBN1	THBS2	NPY
PLN	AEBP1	TIMP3	VCAN	PI3	KLK5
ADIPOQ	RYR3	THBS2	INHBA	CXCL12	COMP
COL5A1	LOX	HOXA5	CTSK	MFAP5	FAP
CNN1	COL5A1	NUAK1	COL1A2	VGLL1	MFAP5
COL6A2	EDNRA	COL5A1	SPARC	CCL11	PI3
COL1A2	NUAK1	SLIT2	AEBP1	EPHB1	PDGFD
DCN	LPL	CXCL12	SERPINE1	OXTR	CHRDL1

The table lists the genes with over-expression indicating higher hazard of death or recurrence, identified by Net-Cox and 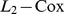 in the consensus ranking across the three datasets.

The intersection of the 60 genes identified by Net-Cox in Table 3 contains 41 unique genes. We performed a literature survey of the 41 genes, out of which eighteen are supported by literature to be related to ovarian cancer shown in Table 4. Most of the genes whose over-expression is associated with poor outcome are stromal or extracellular-related proteins. The genes such as VCAN, TIMP3, THBS2, ADIPOQ, PARC, NPY, MFAP5, DCN, LOX, FBN1, EDNRA, and CXCL12 are either components or modulators of extracellular matrix. In particular, LOX protein is involved in extracellular matrix remodeling by cross-linking collagens. Extracellular matrix remodeling through over-expression of collagens has been shown to contribute to platinum resistance, and platinum resistance is the main factor in chemotherapy failure and poor survival of ovarian cancer patients. Therefore, the identification of these extracellular matrix proteins as biomarkers of early recurrence and poor survival outcome in patients with ovarian cancer is consistent with the suggested pathobiological role of some of these proteins in platinum resistance.

**Table 4:** Literature review of the candidate ovarian cancer genes.

Gene Sym	Reference	Description
ADIPOQ	[30]	ADIPOQ 45T/G and 276G/T polymorphisms is associated with susceptibility to polycystic ovary syndrome(PCOS).
CCL11	[42]	CCL11 signaling plays an important role in proliferation and invasion of ovarian carcinoma cells.
CNN1	[31]	CCN1 plays a role in ovarian carcinogenesis by stimulating survival and antiapoptotic signaling pathways.
CRYAB	[71]	Low expression of lens crystallin CRYAB is significantly associated with adverse ovarian patient survival.
CXCL12	[29]	CXCL12 and vascular endothelial growth factor synergistically induce neoangiogenesis in human ovarian cancers.
DCN	[42]	Ovarian DCN is an ECM-associated component, which acts as a multifunctional regulator of GF signaling in the primate ovary.
EDNRA	[32]	Endothelin peptide is produced before ovulation and the contractile action of EDN2 within the ovary is facilitated via EDNRA.
FBN1	[27]	FBN1 controls the bioactivity of TGF 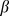 s and associate with polycystic ovary syndrome (PCOS).
IGF1	[41]	Ovarian follicular growth is controlled by the production of intraovarian growth regulatory factors such as IGF1.
INHBA	[40]	INHBA is the promoter of TAF4B; TAF4B in the ovary is essential for proper follicle development.
LOX	[33]	Inhibition of LOX expression portends worse clinical parameters for ovarian cancer.
LPL	[35]	LPL is differentially expressed between preoperative samples of ovarian cancer patients and those of healthy controls.
MFAP5	[36]	MAGP2 is an independent predictor of survival in advanced serous ovarian cancer.
NPY	[72]	NPY receptor is expressed in human primary ovarian neoplasms.
SPARC	[29]	SPARC expression in ovarian cancer cells is inversely correlated with the degree of malignancy.
THBS2	[38]	In ovarian cancer an aberrant methylation process is responsible for down-regulation of THBS2.
TIMP3	[37]	TIMP2 and TIMP3 play functional role in LPA-induced invasion as negative regulators.
VCAN	[28]	VCAN V1 isoform is overexpressed in ovarian cancer stroma compared with normal ovarian stroma and ovarian cancer cells.

This table reports the citations that describe relevance of the signature genes with over-expression indicating higher hazard of death or recurrence, identified by Net-Cox across the three datasets.

### Enriched PPI subnetworks and GO terms

**Figure 7:**
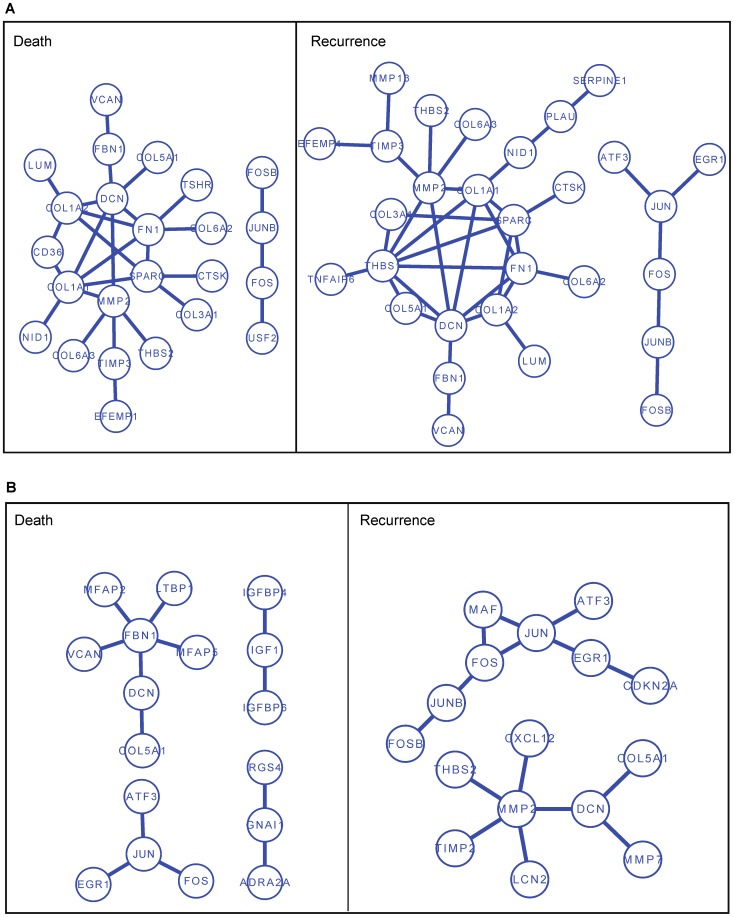
Protein-Protein interaction subnetworks of signature genes identified by Net-Cox on the TCGA dataset. (A) The PPI subnetworks identified by Net-Cox on the co-expression network. (B) The PPI subnetworks identified by Net-Cox on the functional linkage network.

The top-100 signature genes with the largest regression coefficients by Net-Cox and 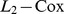 learned from the TCGA dataset were mapped to the human protein-protein interaction (PPI) network obtained from HPRD [43] and also analyzed with DAVID functional annotation tool [44]. We report the densely connected PPI subnetworks constructed from the 100 genes selected by Net-Cox in Figure 7. Compared with the PPI subnetworks generated from the 100 genes selected by 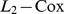, which contain 10 genes in the death subnetwork and 6 genes in the recurrence subnetworks (shown in Figure S4), the subnetworks are both larger and denser. The subnetworks identified from the co-expression networks in Figure 7(A) are also larger than the subnetworks identified by the functional linkage network in Figure 7(B) although many genes are shared. In the recurrence subnetworks, DCN, THBS1, and THBS2 are members of the 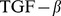 signaling KEGG pathway, and FBN1 controls the bioactivity of TGF
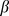
s and relates to polycystic ovary syndrome [27]. In addition, ten genes are members of the focal adhesion KEGG pathway. These results point to a possibility that extracellular matrix signaling through focal adhesion complexes may constitute a pathway by which tumor cells escape chemotherapy and produce recurrence in chemotherapy [45]. Nine genes in the death subnetworks are members of the extracellular matrix(ECM)-receptor interaction KEGG pathway, and eighteen genes are annotated as ECM component. It was shown that ECM acts as a model substratum for the preferential attachment of human ovarian tumor cells in vitro [46]. FOS and JUN constitutes a nuclear signaling components downstream of extracellular signal-regulated kinases (ERK1/2) that are mediators of growth factor and adhesion-related signaling pathways [47]. In addition, the genes are also enriched by regulation of gene expression, positive regulation of cellular process, developmental process, transcription regulator activity, and growth factor binding, all of which are well-known cancer relevant functions. The significantly enriched GO functions are listed in Table S6 and Table S7. Extracellular matrix, extracellular region, and extracellular structure organization are consistently the most significantly enriched in the analysis.

### Laboratory experiment validates FBN1's role in chemo-resistance

**Figure 8:**
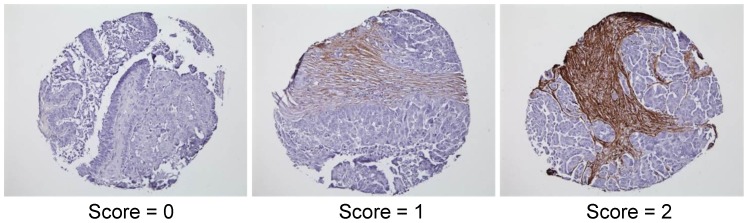
Representative photomicrographs showing various levels of FBN1 expression in ovarian tumor arrays. The brown regions are stromal area showing expression of FBN1.

FBN1 was ranked 1st and 8th by Net-Cox with co-expression network in death and recurrence outcomes while 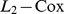 only ranked FBN1 at 27th and 42nd, respectively. It is interesting to note that in the PPI subnetworks in Figure 7(A), FBN1 is connected with VCAN and DCN, both of which bear the annotation of extracellular matrix. The dense subnetwork boosted the ranking of FBN1 when Net-Cox was applied. We further validated the role of FBN1 in ovarian cancer recurrence using tumor microarrays (TMAs) consisting of a cohort of 78 independent patients (see Section **Materials and Methods**). The expression level of FBN1 in ovarian cancer was scored by one observer who is blinded to the clinical outcome and described as: absent (0), moderate (1), and high (2) as illustrated by Figure 8.

In Figure S5A, the Kaplan-Meier survival curve shows the recurrence for groups by the FBN1 staining scores. At the initial 12 month, there is no difference in the recurrence rate between the groups with high and low FBN1 staining. After 12 month, the recurrence rate is lower in the low staining group. The similar patterns are also observed in the re-examination of the gene expression datasets in Figure S5B–E. Except the TCGA dataset on the Affymetrix platform (Figure S5E), the pattern is clearly observed on the other two platforms, exon arrays and Agilent arrays. The discrepancy in the Affymetrix data could be related to data pre-processing or experimental noise. The plots suggest that FBN1 plays a role on platinum-sensitive ovarian cancer, and it could be developed as a target for platinum-sensitive patients with high FBN1 expression after about 12 months of the treatment.

**Figure 9:**
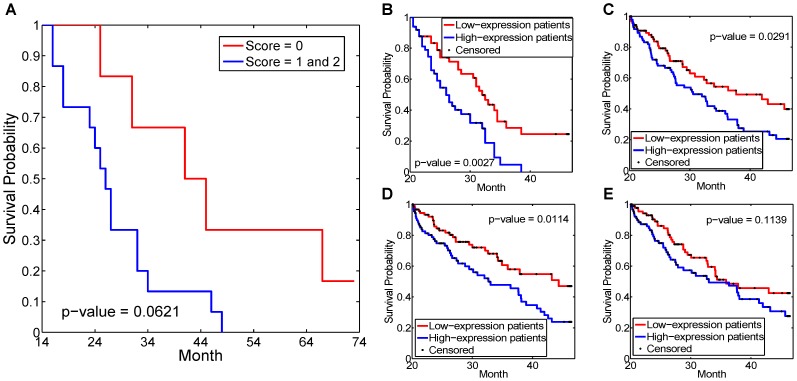
Kaplan-Meier survival plots on FBN1 expression groups. (A) Kaplan-Meier survival curve of recurrence between 14 to 72 month by FBN1 staining groups on Mayo Clinic dataset. (B) Kaplan-Meier survival curve of recurrence between 20 to 72 month by the expression of FBN1 on Tothill dataset. (C)–(E) Kaplan-Meier survival curves of recurrence between 20 to 72 month by the expression of FBN1 on TCGA dataset with AgilentG4502A platform, HuEx-1_0-st-v2 platform, and Affymetrix HG-U133A platform, respectively. In plot(A), the groups with FBN1 staining score 1 and 2 are combined into the high-expression group. In plots(B)–(E), the patients are divided into two groups of the same size by the expression of FBN1.

In the context of ovarian cancer treatment, a platinum-sensitive patient group can be defined as the group of patients who was free of recurrence or developed a recurrence after 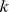 month of the treatment, where 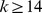 depends on the treatment plan and the follow-up. To better evaluate the role of FBN1, we plot the Kaplan-Meier survival curve only for the platinum-sensitive patients in Figure 9, i.e. we removed all the patients who developed recurrence before 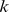 month and considered the follow-ups up to 72 months after the treatment. Due to the small sample size of the Mayo Clinic data, we set 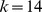 while 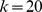 for the gene expression datasets. In Figure 9A, the difference between the survival curves of low FBN1 staining and high staining patient groups is more significant. Similarly, Figure 9B–E show the survival curves for the platinum-sensitive patients for groups by the expression value of FBN1 in gene expression datasets. Compare to the matched curves in Figure S5, the log-rank test 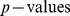 are more significant except the TCGA dataset on the Affymetrix platform. Overall, the observations strongly support the hypothesized role of FBN1 in platinum-sensitive ovarian cancer patients.

## Discussion

Many methods were proposed for survival analysis on high-dimensional gene expression data with highly correlated variates [4], [5]. In this paper, we propose Net-Cox, a network-based survival model, which to our knowledge is among the first models that directly incorporate network information in survival analysis. The graph Laplacian constraint introduced in Net-Cox is positive definite and thus, the Net-Cox model can be solved as efficiently as solving the 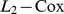 model. In the dual form of Net-Cox, the model is scalable to genomic data with 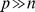. Net-Cox not only makes survival predictions but also generate densely connected subnetworks enriched by genes with large regression coefficients.

Net-Cox is most related to the 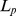 shrinkage-based Cox models typically with 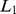 (Lasso) and 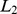 (ridge) penalties [5]. The purpose of applying 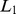 regularization is to obtain a sparse estimate of the linear coefficients for solving the high-dimensionality problem. A Ridge penalty results in small regression coefficients to avoid overfitting problem with the small sample size. Compared with Net-Cox, neither Lasso nor ridge regularized Cox regression models are designed to incorporate any prior information among genes in the objective function for survival analysis. Another alternative solution in the literature is to apply dimension reduction methods to obtain a small number of features for subsequent survival analysis such as principal components analysis (PCA) [48]–[50] and partial least squares (PLS) [51]–[54]. These methods first compute the principle components to capture the maximal covariance with the outcomes or the maximal variance in the gene expression data, and then project the original high-dimensional gene expressions into a space of the directions of the principle components. Typically, these methods do not utilize any prior information. It is also usually difficult to interpret the results since the features in the project space are not directly mappable to any particular gene expression. There are also tree-based ensemble methods for survival analysis such as bagging of survival trees and random forests [55], [56]. The tree-based methods usually also require a variable selection step to reduce the dimensionality. Multiple trees are then built from different samplings of training data and the results of the individual trees are aggregated for making predictions. Since the trees are built from random sampling, the resulted forests consist of different trees. Thus, the interpretation of the trees can be very difficult [4].

In [57], a supervised group Lasso approach (SGLasso) is proposed to account for the cluster structure in gene expression data as prior information in survival analysis. In this approach, gene clusters are first identified with clustering. Important genes are then identified with Lasso model within each cluster and finally, the clusters are selected with group Lasso. More recently, the method in [58] combined a group Lasso constraint with Lasso Cox regression (sparse-group Lasso). An additional parameter is introduced to balance between Lasso and group Lasso constraints. There are two major discrepancies between Net-Cox and the graph Lasso methods. First, while group Lasso assumes non-overlapping cluster structures among gene expressions, the gene network introduced in Net-Cox captures more global relation among all the genes. Specifically, beyond the cluster partition of genes into co-expression groups, a gene network represents pair-wise relationships between genes, which contain information of modularities, subgraph structures and other global properties such as centralities and closenesses. Second, while SGLasso adopts an unsupervised strategy to cluster genes as predefined groups for selection, Net-Cox identifies subnetwork signatures in a supervised manner, in which the selected subnetworks are enriched by genes with large regression coefficients by the design of the network constraint. In Table S3(g), we reported the results of group Lasso and sparse-group Lasso in the five-fold cross-validation with the R package “SGL” [58]. Compared with the CVPLs by the other methods in Table S3(a)–(f), the CVPLs in Table S3(g) for group Lasso and sparse-group Lasso are consistently lowest when 25 or 100 gene clusters are used as groups. Thus, we did not further compare and analyze other results by the group Lasso models.

The experiments in this paper clearly demonstrated that the network information is useful for improving the accuracy of survival prediction as well as increasing the consistency in discovering signature genes across independent datasets. Since the signature genes were discovered based on their relation in the networks, they enrich dense PPI subnetworks, which are useful for pathway analysis. It is also interesting to note that the PPI subnetworks of signature genes identified by Net-Cox on the TCGA dataset is enriched by extracellular matrix proteins such as collagens, fibronectin, and decorin. Previous gene expression studies had identified stromal gene signatures in ovarian tumors to be associated with poor survival outcome [24]. Therefore, our observation that the stromal subnetwork enriched by extracellular matrix proteins and stromal-related proteins is consistent with the role of stromal gene signature in poor prognosis. Finally, collagen matrix remodelling has been linked to platinum resistance, and ovarian cancer cells grown on collagens are more resistant to platinum agents than their counterpart grown on non-collagen substratum [59]. The tumor array validation indicates that FBN1 can serve as a biomarker for predicting recurrence of platinum-sensitive ovarian cancer.

## Materials and Methods

This section describes the data preparation, the Cox models and the experimental setup. We first describe the construction of the gene relation networks and the processing of the microarray gene expression datasets. We then review the Cox regression models and introduce the regularization framework of Net-Cox by adding a network constraint to the Cox model. The algorithms to efficiently estimate the optimal solution for Net-Cox are outlined. We also describe the procedures for cross-validation and parameter tuning, and the evaluation measures. At last, tumor array preparation is explained.

### Gene relation network construction

We denote gene relation network by 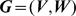, where 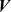 is the vertex set, each element of which represents a gene, and 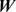 is a 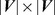 positively weighted adjacency matrix. 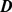 is a diagonal matrix with 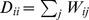 and 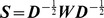 is the normalized weighted adjacency matrix by dividing the square root of the column sum and the row sum. Two gene relation networks were used with Net-Cox, the gene co-expression network and the gene functional linkage network.

#### Gene co-expression network

A gene co-expression network was generated from a gene correlation graph model. In the weighted adjacency matrix 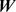, each 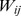 is the reliability score [60] based on the absolute value of the Pearson's correlation coefficients between genes 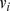 and 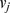, calculated as 
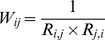
, where 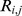 is gene 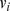
's rank among all the genes with respect to the correlation with gene 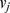 and 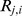 is gene 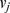
's rank with respect to the correlation with gene 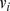. Note that the gene co-expression network is directly inferred from the gene expression dataset. Thus, a gene co-expression network is specific to the dataset used for computing the co-expression network.

#### Gene functional linkage network

A human gene functional linkage network was constructed by a regularized Bayesian integration system [61]. The network contains maps of functional activity and interaction networks in over 200 areas of human cellular biology with information from 30,000 genome-scale experiments. The functional linkage network summarizes information from a variety of biologically informative perspectives: prediction of protein function and functional modules, cross-talk among biological processes, and association of novel genes and pathways with known genetic disorders [61]. Each edge in the network is weighted between [0,1] to quantify the functional relation between two genes. Thus, the functional linkage network provides much more comprehensive information than Human protein-protein interaction network, which was more frequently used as the network prior knowledge.

### Gene expression dataset preparation

Three independent microarray gene expression datasets for studying ovarian carcinoma were used in the experiments [3], [24], [25]. The information of patient samples in each dataset is given in Table 1. All the three datasets were generated by the Affymetrix HG-U133A platform. The raw .CEL files of two datasets were downloaded from GEO website (Tothill: GSE9899) and (Bonome: GSE26712) [24], [25]. The TCGA dataset was downloaded from The Cancer Genome Atlas data portal [3]. The raw files were normalized by RMA [62]. After merging probes by gene symbols and removing probes with no gene symbol, a total of 7562 unique genes were derived from the 22,283 probes and overlapped with the functional linkage network for this study. Note that the Bonome dataset does not provide information on recurrence. Thus, only TCGA and Tothill datasets were used for studying recurrence while all the three datasets were used for studying death. In cross-dataset validation, the batch effects among the three datasets were removed by applying ComBat [63]. Besides testing all the genes, for a better focus on genes that are more likely to be cancer relevant, we derived a set of 2647 genes from the cancer gene list compiled by Sloan-Kettering Cancer Center (SKCC) [64].

The TCGA datasets with AgilentG4502A platform (gene expression array) and HuEx-1_0-st-v2 (exon expression array) were used to evaluate the signature gene FBN1 in Figure 9. The processed level 3 data with expression calls for gene/exon were downloaded from the TCGA data portal.

### Cox proportional hazard model

Consider the Cox regression model proposed in [6]. Given 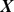, the gene expression profile of 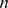 patients over 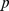 genes, the instantaneous risk of an event at time 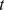 for the 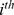 patient with gene expressions 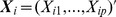 is given by
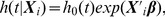
(1)where 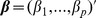 is a vector of regression coefficients, and 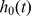 is an unspecified baseline hazard function. In the classical setting with 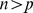, the regression coefficients are estimated by maximizing the Cox's log-partial likelihood:
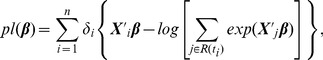
(2)where 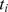 is the observed or censored survival time for the 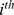 patient, and 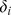 is an indicator of whether the survival time is observed (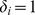) or censored (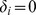). 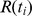 is the risk set at time 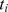, i.e. the set of all patients who still survived prior to time 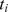. The commonly used Breslow estimator [65] to estimate the baseline hazard 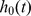 is given by
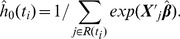
(3)The partial likelihood and the Breslow estimator are induced by the total log-likelihood
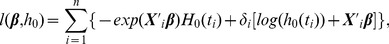
(4)with
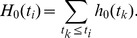
(5)The optimal regression coefficients 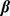 is estimated based on the maximization of the total log-likelihood by alternating between maximization with respect to 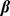 (with Newton-Raphson) and 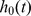 (by equation (3)).

In the analysis of microarray gene expressions, the number of gene features 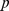 is larger than the number of subjects 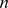 by several magnitudes (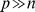). Fitting the Cox regression model will lead to large regression coefficients, which are not reliable. One possible solution is to introduce a 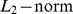 constraint to shrink regression coefficients estimates towards zero [7], [10]. In the 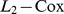 model, the regression coefficients are estimated by maximizing the penalized total log-likelihood:
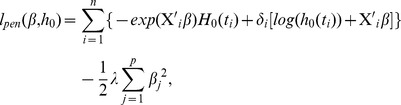
(6)where 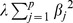 is the penalty term and 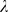 is the parameter controlling the amount of shrinkage. Another possibility is to introduce a 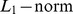 constraint for variable selection [11], [13]. The 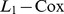 model penalizes the log-partial likelihood (equation (2)) by 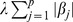 leading to:
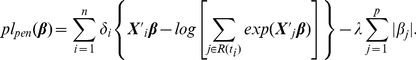
(7)In our experiments, R package “glmnet” [66] was used in the implementation of 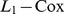.

### Network-constrained Cox regression (Net-Cox)

We introduce a network-constraint to the Cox model as follows,
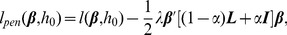
(8)where 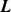 is a positive semidefinite matrix derived from network information, 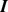 is an identity matrix, and 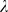 is the parameter controlling the weighting between the total likelihood and the network constraint. 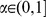 is another parameter weighting the network matrix and the identity matrix in the network constraint. For convenience, we define 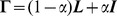 and rewrite the object function as
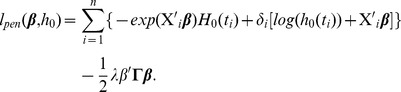
(9)The term 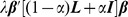 in equation (8) is a network Laplacian constraint to encode prior knowledge from a network. Given a normalized graph weight matrix 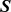, we assume that co-expressed (related) genes should be assigned similar coefficients by defining the following cost term over the coefficients,
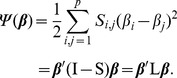
(10)As illustrated in Figure 1, the Laplacian constraint encourages a smoothness among the regression coefficients in the network. Specifically, for any pair of genes connected by an edge, there is a cost proportional to both the difference in the coefficients and the edge weight. Large difference between coefficients on two genes connected with a highly weighted edge will result in a large cost in the objective function. Thus, the objective function encourages assigning similar weights to genes connected by edges of larger weights. By adding an additional 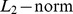 constraint to 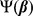 weighted by 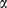, we obtain the network constraint 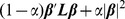 = 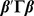 in equation (8) and (9). The 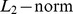 of 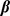 similarly regularizes the uncertainty in the network constraint, which could have a singular Hessian matrix, and the 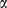 parameter balances between the 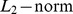 and the “Laplacian-norm”. The smaller the 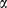 parameter, the more importance put on the network information.

### Alternating optimization algorithm

The objective function defined by equation (9) can be solved by alternating optimization of 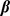 and 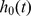. The maximization with respect to 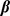 is done by Newton-Raphson method. The derivative of equation (9) is
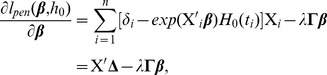
(11)where 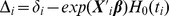, and the second derivative is
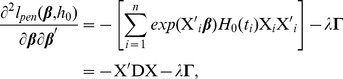
(12)where 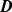 is the diagonal matrix with 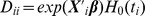. Thus, the full algorithm to solve the Net-Cox model is given below.

1. **Initialization:** 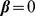; Compute 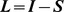.
2. **Do** until convergence
  - **Do** Newton-Raphson iteration
    - Compute the first derivative 
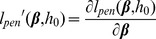

    - Compute the second derivative 
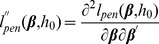

    - Update 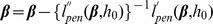

  - Update 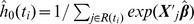

3. **Return** 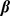

Using Newton-Raphson method to update 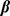 requires inverting the Hessian matrix, which is time consuming and often inaccurate. An alternative approach is to reduce the covariant space from *p* to *n*, which relates to singular value decomposition that exploits the low rank of the gene expression matrix 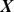 [10]. The equation
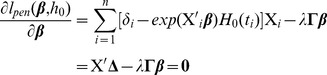
(13)implies that 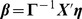 for some 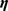. Thus, the dual form of equation (9) with respect to 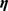 is
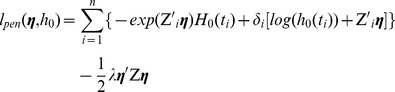
(14)with 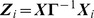 and 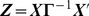. In its dual form, it is clear that the new object function (14) is equivalent to equation (9) but the problem dimension is reduced from 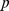 to 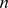.

### Cross validation and parameter tuning

To determine the optimal tuning parameters 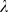 and 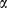, we performed five-fold cross-validation following the procedure proposed by [10] on each of the three datasets. In the cross-validation, four folds of data are used to build a model for validation on the fifth fold, cycling through each of the five folds in turn, and then the 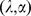 pair that maximizes the cross-validation log-partial likelihood (CVPL) are chosen as the optimal parameters. CVPL is defined as
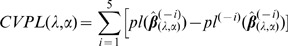
(15)where 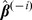 is the optimal 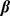 learned from the data without the 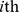 fold. In the equation, 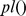 denotes the log-partial likelihood on all the samples and 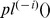 denotes the log-partial likelihood on samples excluding the 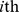 fold. We performed a grid search for the optimal 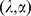 maximizing the sum of the contributions of each fold to the log-partial likelihood in CVPL. In particular, 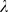 was chosen from 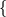
1e-5, 1e-4, 1e-3, 1e-2, 1e-1, 1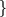 (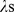 larger than 1 do not change the ranking of 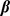 anymore), and 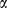 was chosen from 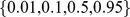. Note that, when 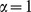, Net-Cox ignores the network information and is reduced to 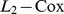. For 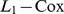, the optimal 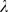 was chosen from 1000 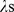 by the “glmnet” parameter setting with the largest CVPL.

### Evaluation measures

The Log-rank test [67] and time-dependent ROC [68] were used to evaluate measurements of the prediction performance by a survival model. For the gene expression profile 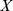 in the test set, the prognostic indexes 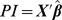 is computed, where 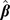 is the regression coefficients of the survival model, to rank the patients by descending order. We assigned the top 40% of the patients as the 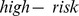 group and the bottom 40% as the 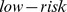 group.

The Log-rank test is a statistical hypothesis test for comparison of two Kaplan-Meier survival curves with the null hypothesis that there is no difference between the population survival curves, i.e. the probability of an event occurring at any time point is the same for each population. The test statistic is compared with a 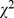 distribution with one degree of freedom to derive the significance 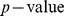 reflecting the difference between two survival curves. The log-rank test only evaluates whether the patients are assigned to the “right group” but not how well the patients are ranked within the group by examining the 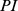. A more refined approach is afforded by the time-dependent ROC curves [68], [69]. Time-dependent ROC curves evaluate how well the 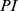 classifies the patients into 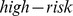 and 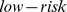 prognosis groups. Letting 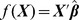, we can define time-dependent sensitivity and specificity functions at a cutoff point 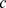 as
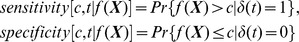
with 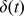 being the event indicator at time 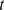 [69]. The corresponding ROC curve for any time 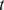, 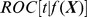, is the plot of 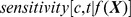 versus 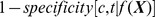 with different cutoff point 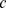. 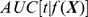 is denoted as the area under the 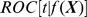 curve. A larger 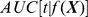 indicates better prediction of time to event at time 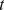, as measured by sensitivity and specificity evaluated at time 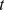. We plot the AUCs at each time 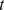 to compare the methods.

To select gene variables in the multi-variate scenario by Net-Cox and 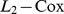, we ranked the genes by the magnitude of the coefficients 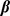. To justify this simple ranking method, we examined the relation between the magnitude of the coefficients for each gene and the contribution of the gene to the log-partial likelihood in Figure S6. It is clear in the plot that the genes towards the two tails of the ranking list contributes most of the likelihood, and the proportion of the contributions are consistent with the ranking. For 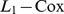, we ranked the genes by the first-time jump into the active set when decreasing the tuning parameter 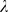 in the solution path.

### Tumor array preparation

With approval by the Mayo Clinic Institutional Review Board, archived ovarian epithelial tumor specimens from patients with advanced-stage, high-grade serous, or endometrioid tumors obtained prior to exposure to any chemotherapy were utilized to construct the TMA array. The array was constructed using a custom-fabricated device that utilizes a 0.6-mm tissue corer and a 240-capacity recipient block. Triplicate cores from each tumor were included, as were cores of liver as fiducial markers and controls for immunohistochemistry reactions. Five-micrometer-thick sections were cut from the TMA blocks. Immunohistochemistry was performed essentially as described in [70]. Sections of tissue arrays were deparaffinized, rehydrated, and submitted to antigen retrieval by a steamer for 25 minutes in target retrieval solution (Dako, Carpinteria, CA, USA). Endogenous peroxide was diminished with 3% 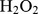 for 30 min. Slides were blocked in protein block solution for 30 min and then blocked with avidin and biotin for 10 min each, followed by overnight incubation with 1∶1000 diluted Anti-FBN1 antibody (HPA021057, Sigma-Aldrich) at 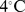. The sections were then incubated with biotinylated universal link for 15 min and streptavidin for 25 min at 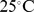. Slides were developed in diaminobenzine and counterstained with hematoxylin.

## Supporting Information

Figure S1 **Time-dependent AUCs averaged across the five test folds in five-fold cross-validation.** The plots report the results of using Sloan-Kettering cancer genes (left column) and all mappables genes (right column). The plots show the results for the death outcome of TCGA dataset (A), the death outcome of Tothill dataset (B), the death outcome of Bonome dataset (C), the tumor recurrence outcome of TCGA dataset (D) and the tumor recurrence outcome of Tothill dataset (E).
(PDF)

Figure S2 **Marker gene consistency (all mappable genes).** The x-axis is the number of selected signature genes ranked by each method. The y-axis is the percentage of the overlapped genes between the selected genes across the ovarian cancer datasets. The results are shown for the death outcome (A) and the tumor recurrence outcome (B).
(PDF)

Figure S3
**Cross-dataset survival prediction (all mappable genes).** The first four columns of plots show the Kaplan-Meier survival curves for the two risk groups defined by Net-Cox (co-expression network), Net-Cox (functional linkage network), 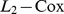 and 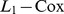. The fifth column of plots compare the time-dependent area under the ROC curves based on the estimated risk scores (PIs). The results are shown for the death outcome by training with TCGA dataset and test on Tothill Dataset (A), for the death outcome by training with TCGA dataset and test on Bonome Dataset (B) and for the tumor recurrence outcome by training with TCGA dataset and test on Tothill Dataset (C).(PDF)

Figure S4
**Protein-Protein interaction sub-networks of marker genes identified by Net-Cox and** 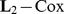 **on the TCGA dataset.** (A) The PPI subnetworks identified by Net-Cox on the co-expression network. (B) The PPI subnetworks identified by Net-Cox on the functional linkage network. (C) The PPI subnetwrks identified by 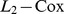.(PDF)

Figure S5 **Kaplan-Meier survival plots on FBN1 expression groups.** (A) Kaplan-Meier survival curve of recurrence by FBN1 staining groups. The group with low FBN1 expression has a lower recurrence rate compared with the groups with high expression after 12 month of treatment. (B) Kaplan-Meier survival curve of recurrence by the expression of FBN1 on Tothill dataset. (C)–(E) Kaplan-Meier survival curves of recurrence by the expression of FBN1 on TCGA dataset with AgilentG4502A platform, HuEx-1_0-st-v2 platform, and Affymetrix HG-U133A platform, respectively. In plots(B)–(E), the patients are divided into two groups of the same size by the expression of FBN1.
(PDF)

Figure S6 **Contributions to the log-partial likelihood by each individual gene by Net-Cox on the Tothill dataset (Sloan-Kettering cancer genes).** The x-axis is the index of the genes sorted by coefficients.
(PDF)

Table S1 **Optimal parameters of Net-Cox.** The parameters are selected by CVPLs in five-fold cross-validation. (a) Sloan-Kettering cancer genes. (b) All mappable genes.
(PDF)

Table S2
**Statistical significance of the improvement in time-dependent AUCs in cross-dataset evaluation (Sloan-Kettering cancer genes).** The R package “timeROC” (the algorithm was described in the paper “Estimating and Comparing time-dependent areas under ROC curves for censored event times with competing risks”) was used to compute the 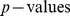. The null hypothesis asserts that two time-dependent AUCs estimated by two models are equal. The significant 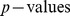 smaller than 0.1 are bold. The tables show the results for the death outcome by training with TCGA dataset and test on Tothill Dataset (a), for the death outcome by training with TCGA dataset and test on Bonome Dataset (b), for the tumor recurrence outcome by training with TCGA dataset and test on Tothill Dataset (c).(PDF)

Table S3
**Cross validation partial likelihood (CVPL) in five-fold cross-validation (Sloan-Kettering cancer genes).** (a) The death outcome of TCGA dataset. (b) The tumor recurrence outcome of TCGA dataset. (c) The death outcome of Tothill dataset. (d) The tumor recurrence outcome of Tothill dataset. (e) The death outcome of Bonome dataset. (f) 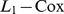. (g) Group Lasso and Sparse-Group Lasso.(PDF)

Table S4
**Log-rank test** 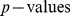 **of the test folds on five-fold cross-validation.** The most significant 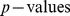 across four models with cut-off 0.05 are bold. (a) Sloan-Kettering cancer genes and the death outcome. (b) Sloan-Kettering cancer genes and the tumor recurrence outcome. (c) All mappable genes and the death outcome. (d) All mappable genes and the tumor recurrence outcome.(PDF)

Table S5
**Log-rank test** 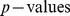 **in cross-dataset evaluation (all mappable genes).** The survival prediction performance on Tothill and Bonome datasets using the Cox models trained with TCGA dataset are reported.(PDF)

Table S6
**Enriched GO terms by the signature genes of death outcome.** The 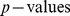 in 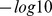 scale are shown for the enriched GO terms.(PDF)

Table S7
**Enriched GO terms by the signature genes of recurrence.** The 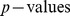 in 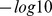 scale are shown for the enriched GO terms. A “X” denotes a 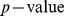 larger than 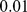.(PDF)
