# Supplementary figures and images for: Network-based Survival Analysis Reveals Subnetwork Signatures for Predicting Outcomes of Ovarian Cancer Treatment
Source: PLoS Comput Biol. 2013 Mar 21;9(3):e1002975. doi: 10.1371/journal.pcbi.1002975 (PMC3605061; doi:10.1371/journal.pcbi.1002975)

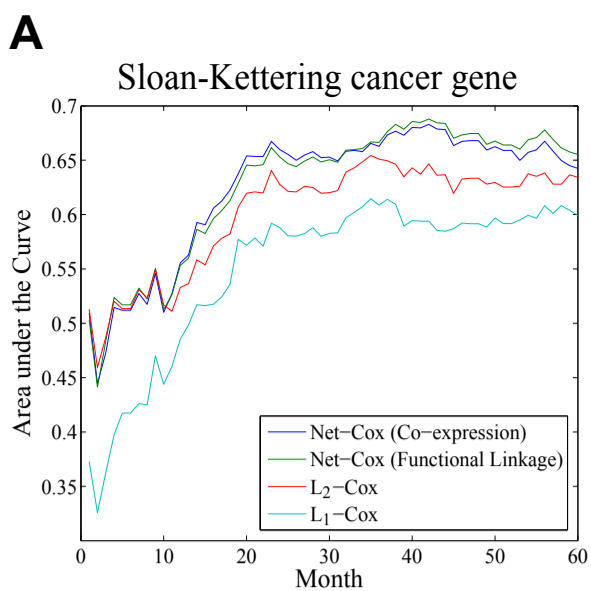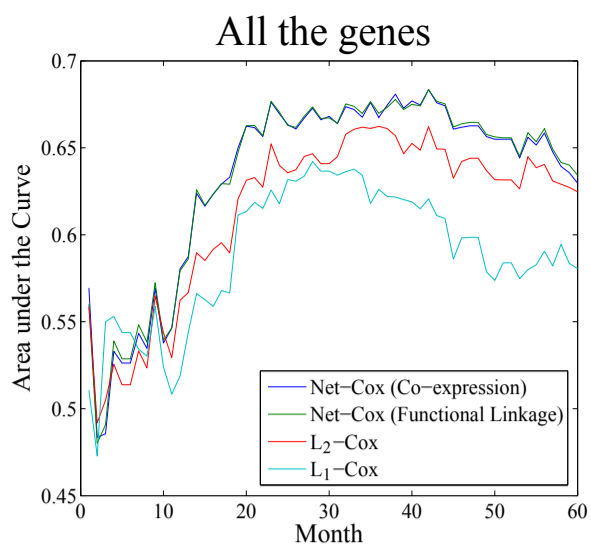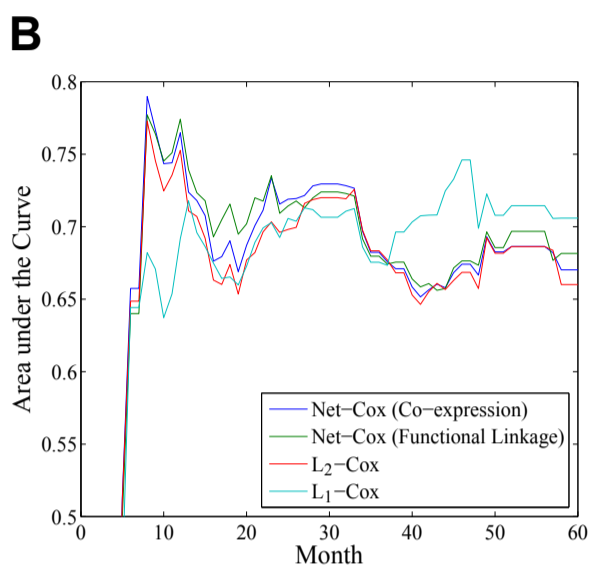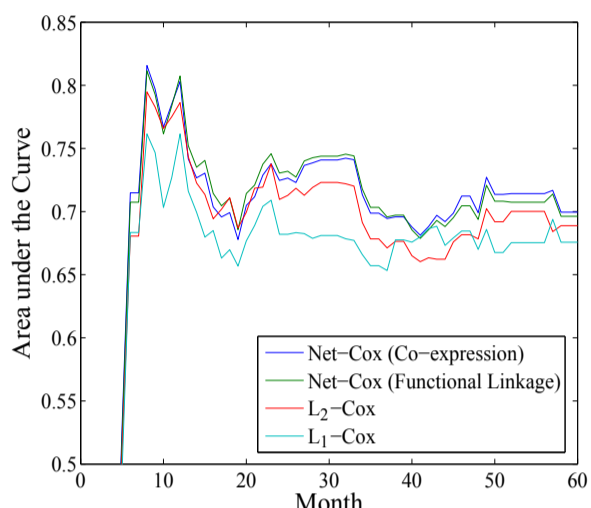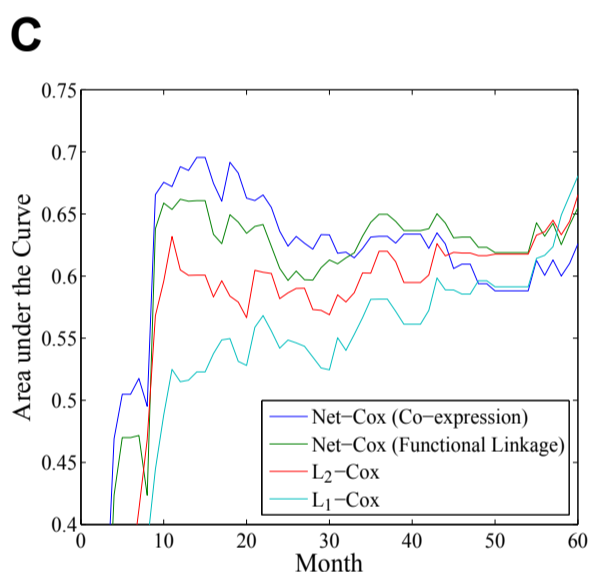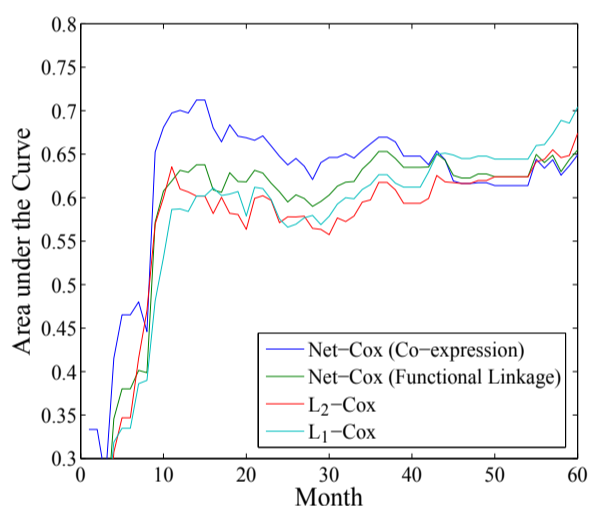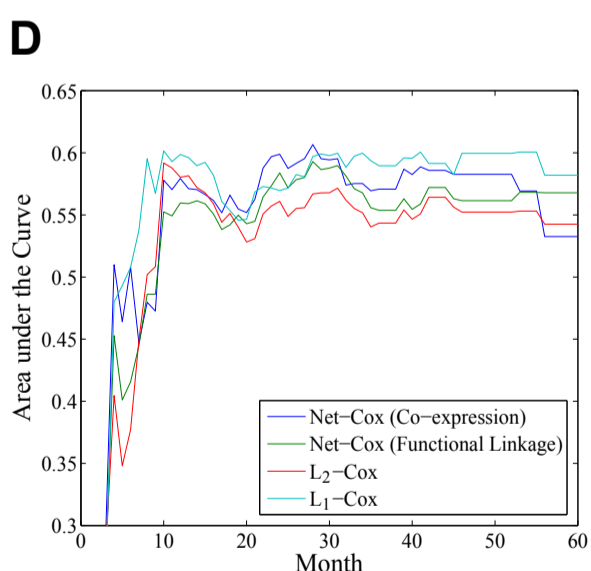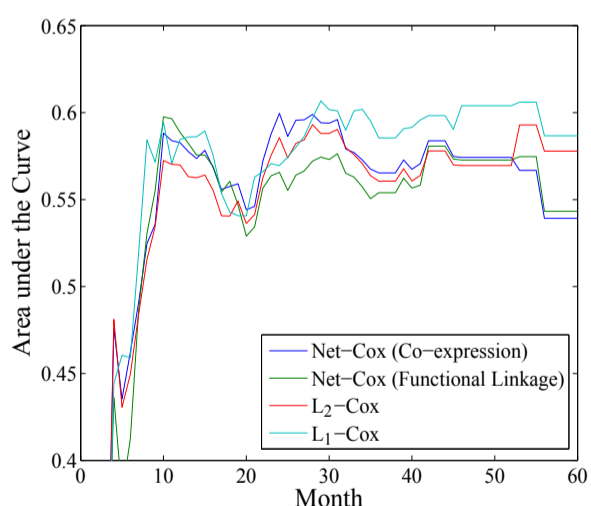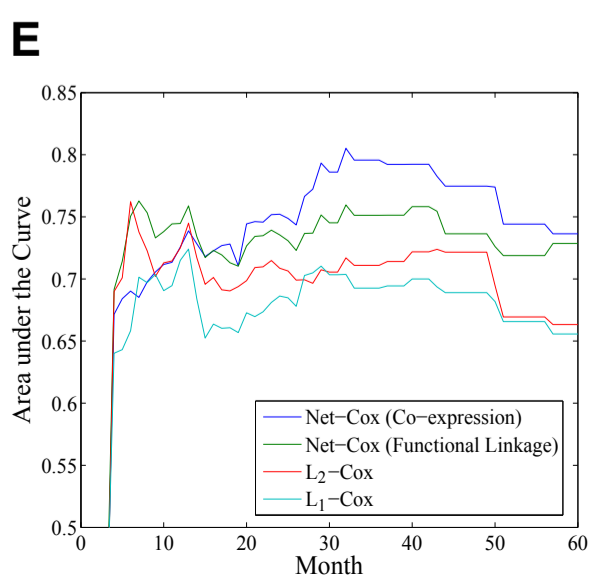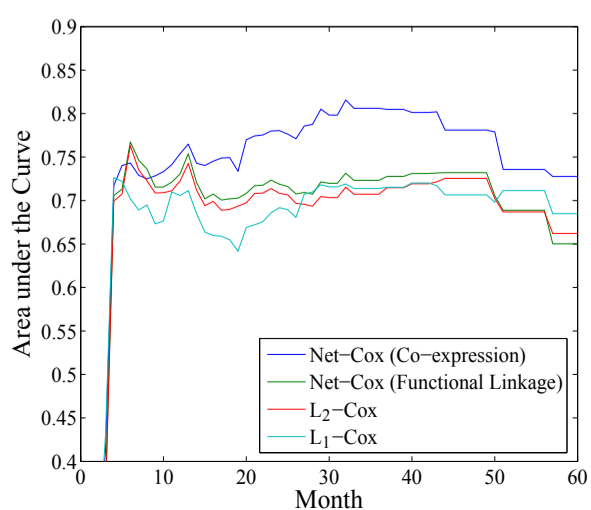

Supplement: Figure S1 — Time-dependent AUCs averaged across the five test folds in five-fold cross-validation. The plots report the results of using Sloan-Kettering cancer genes (left column) and all mappables genes (right column). The plots show the results for the death outcome of TCGA dataset (A), the death outcome of Tothill dataset (B), the death outcome of Bonome dataset (C), the tumor recurrence outcome of TCGA dataset (D) and the tumor recurrence outcome of Tothill dataset (E). (PDF) [file pcbi.1002975.s001.pdf]

**A**

Percentage of Overlapped Genes

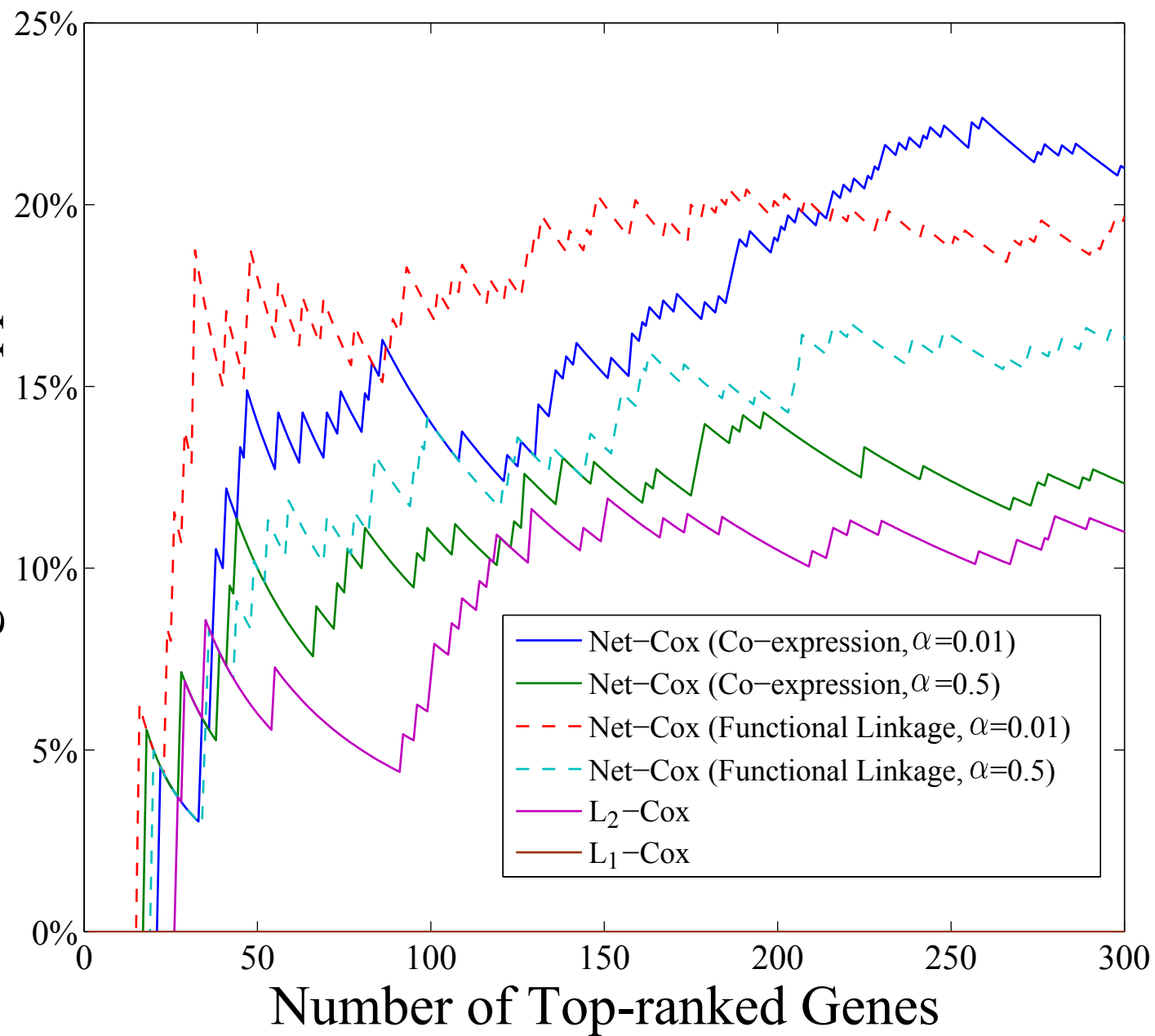**B**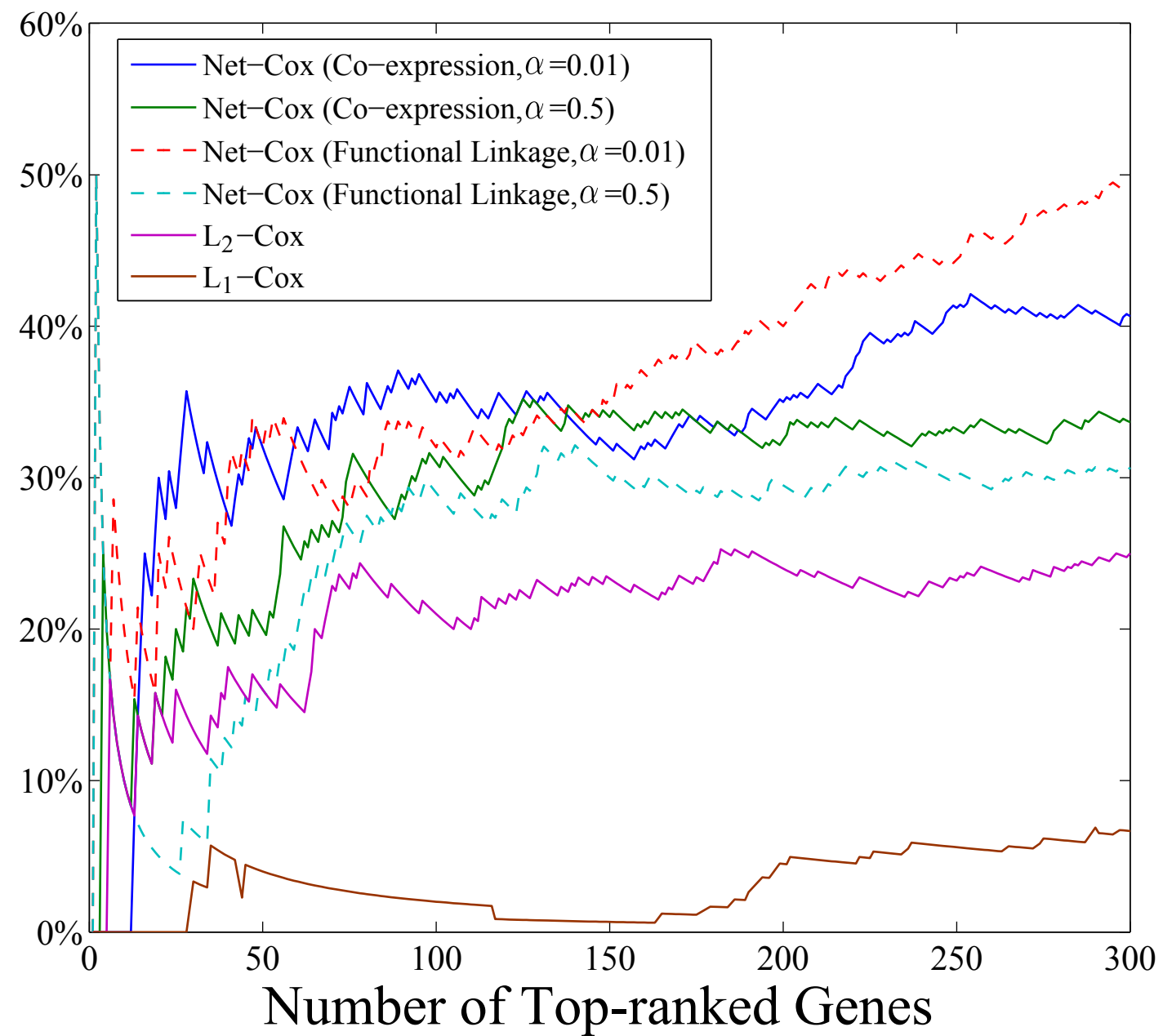

Supplement: Figure S2 — Marker gene consistency (all mappable genes). The x-axis is the number of selected signature genes ranked by each method. The y-axis is the percentage of the overlapped genes between the selected genes across the ovarian cancer datasets. The results are shown for the death outcome (A) and the tumor recurrence outcome (B). (PDF) [file pcbi.1002975.s002.pdf]

**A**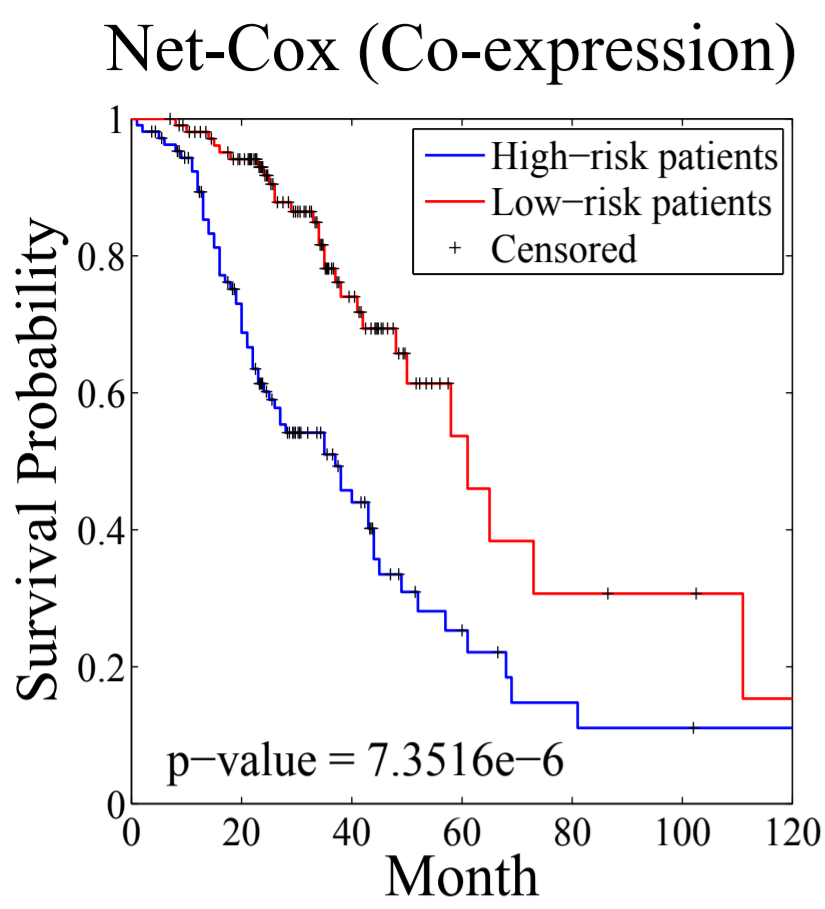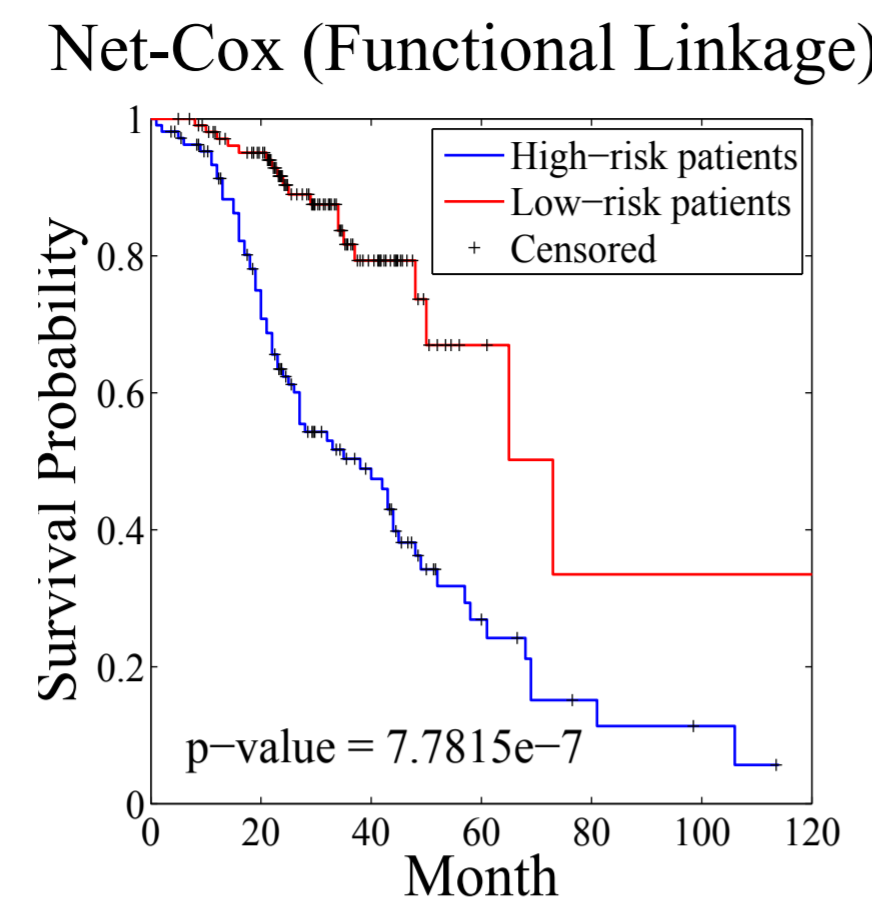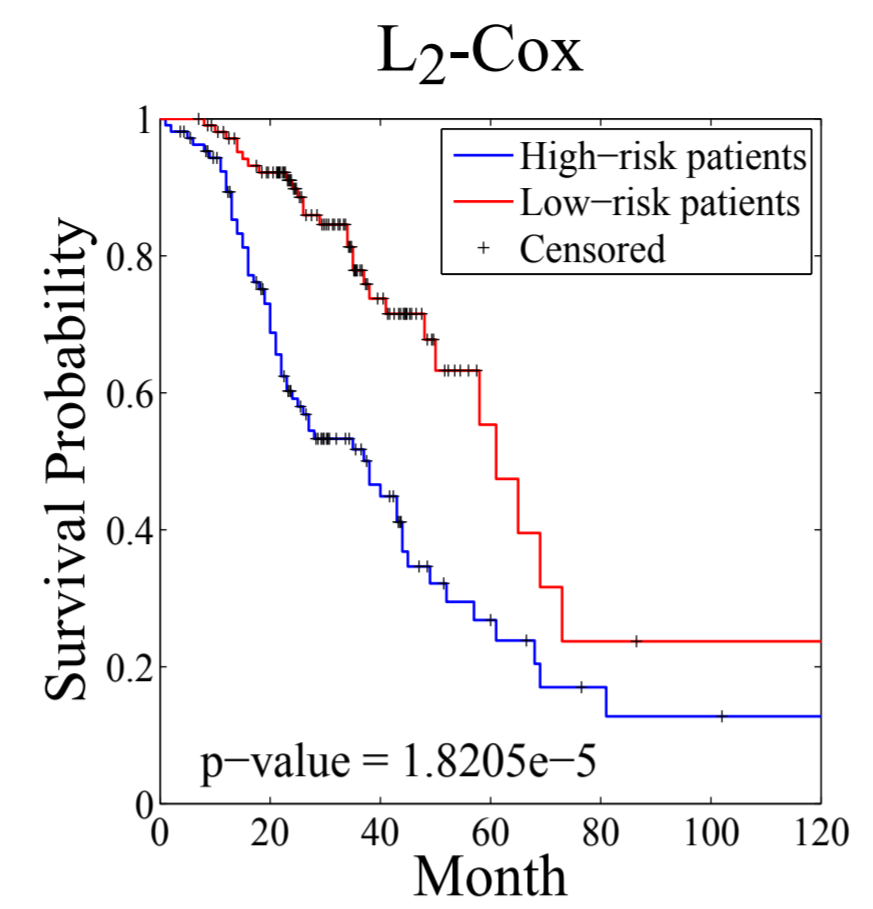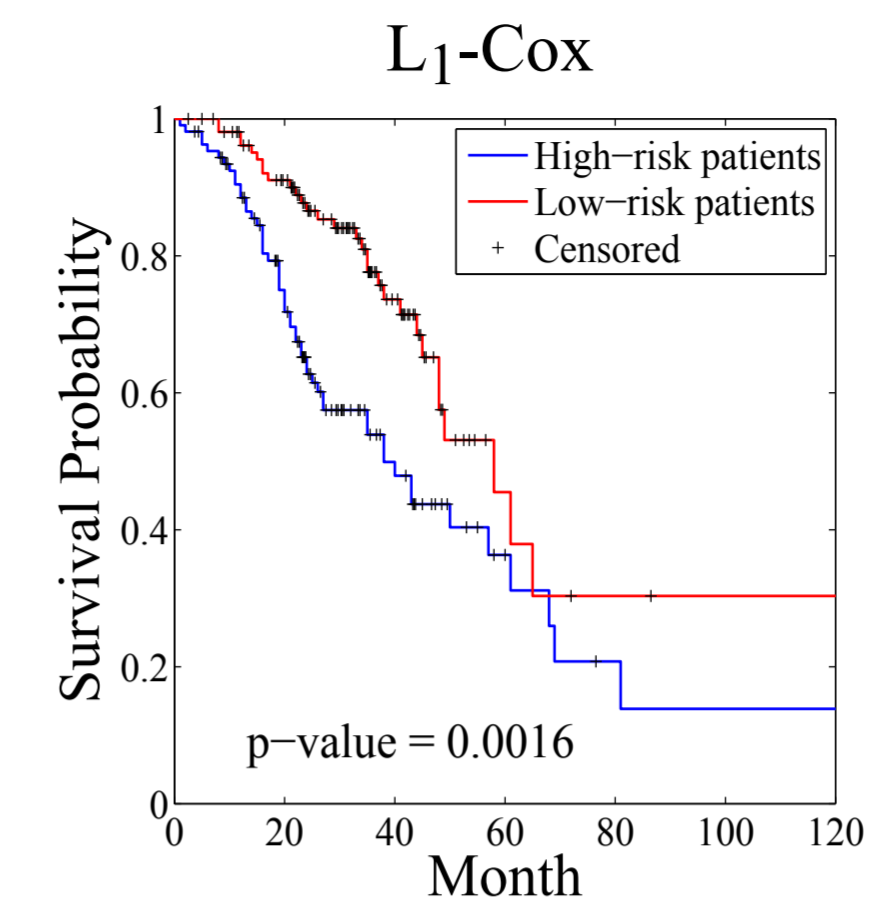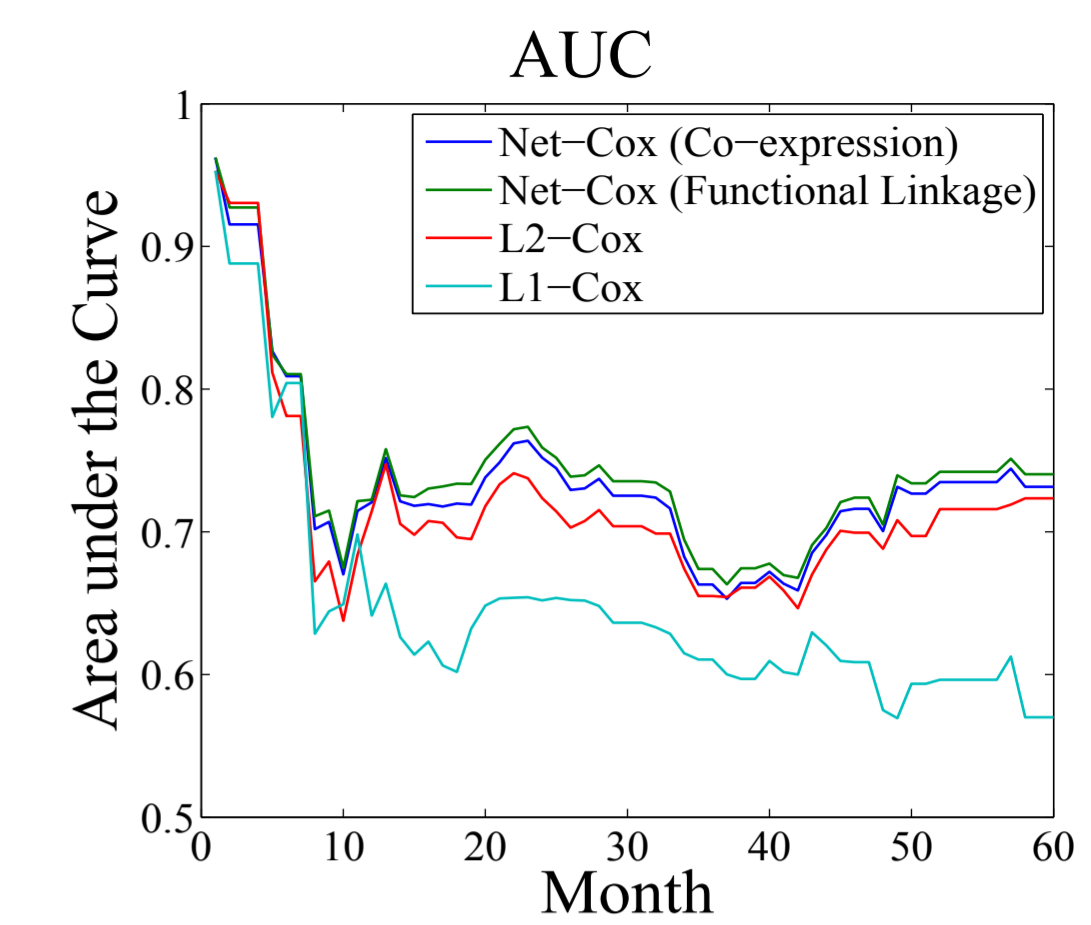**B**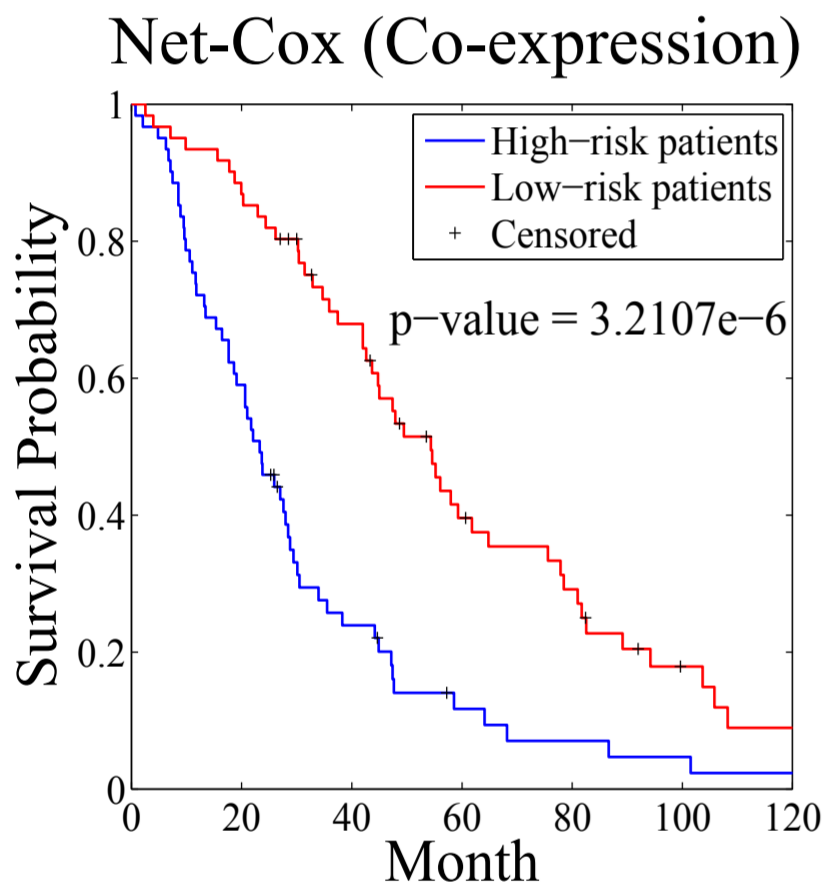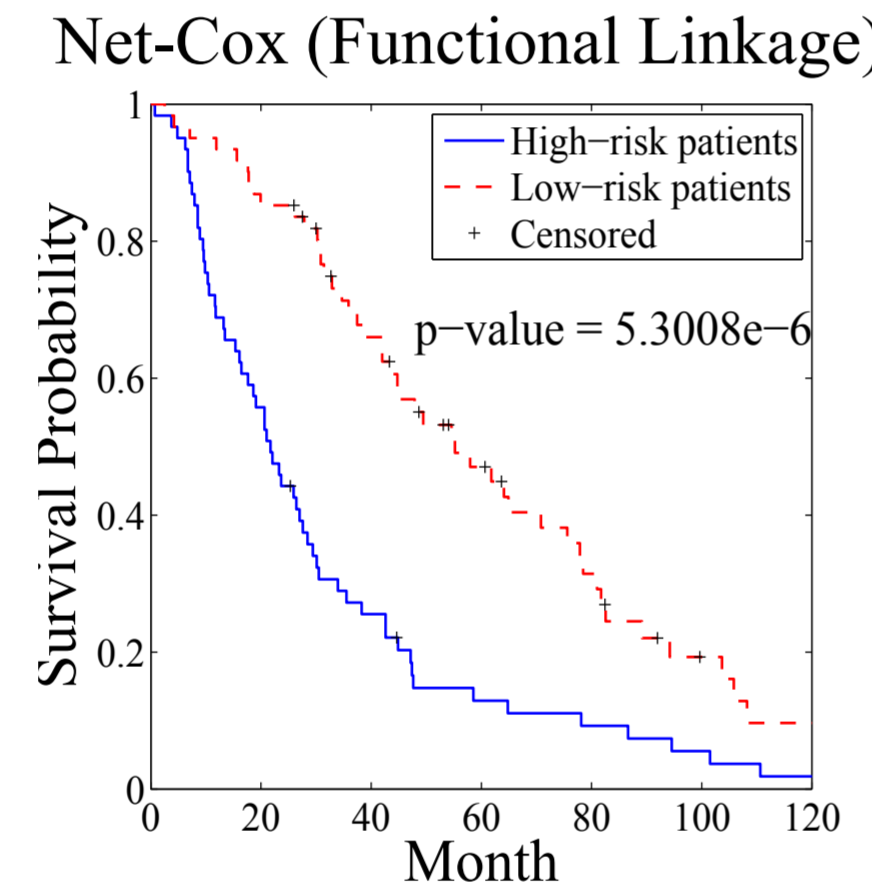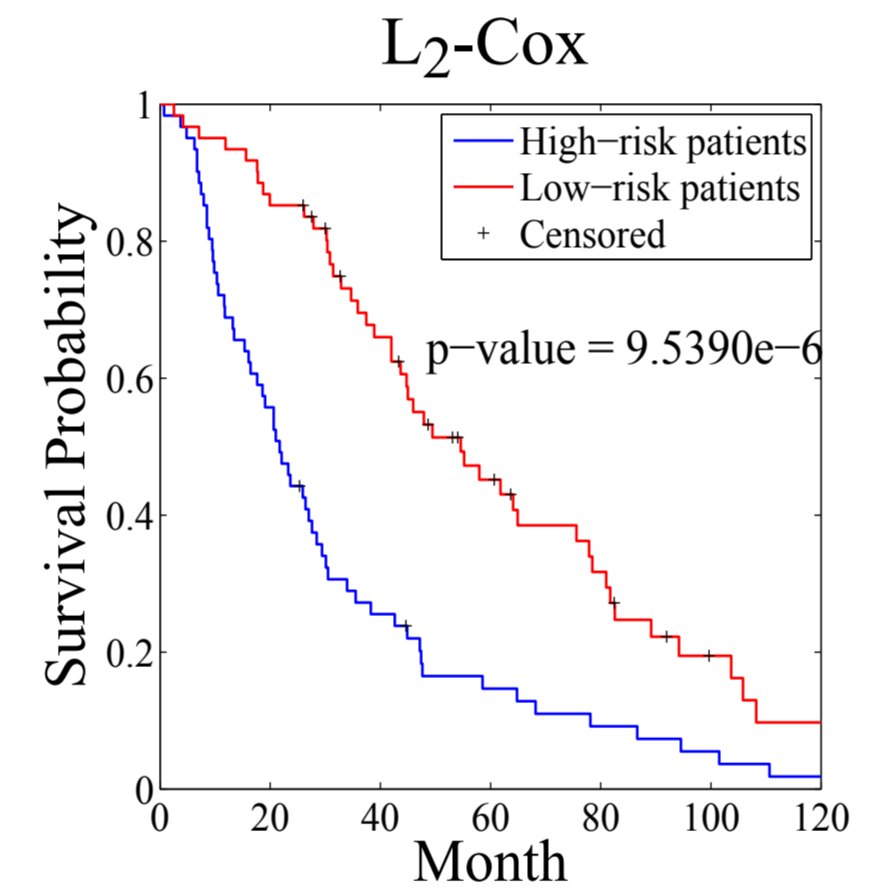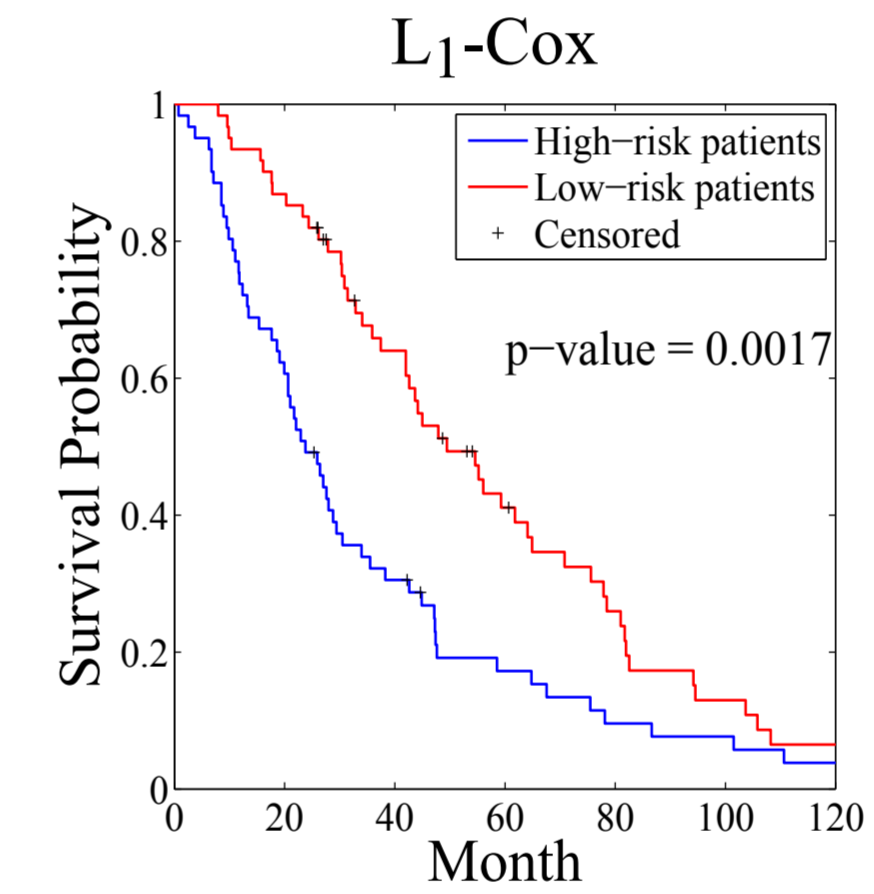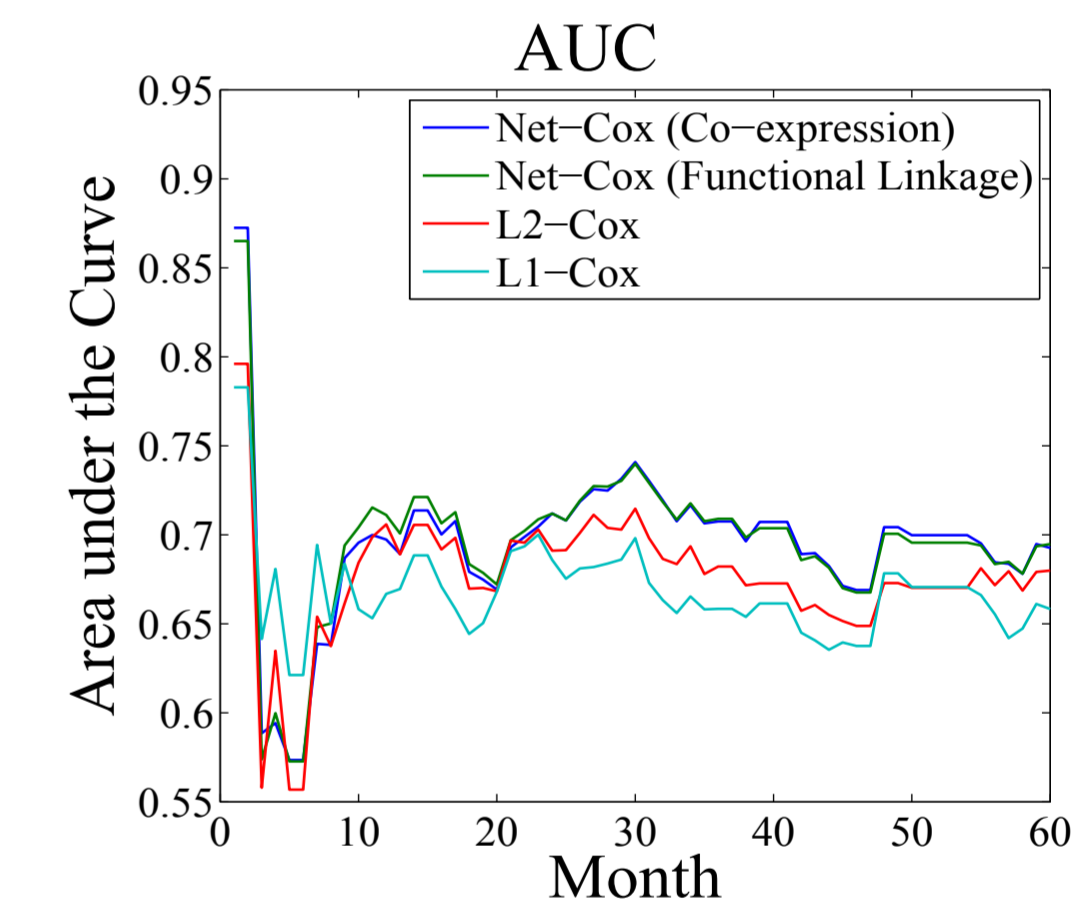**C**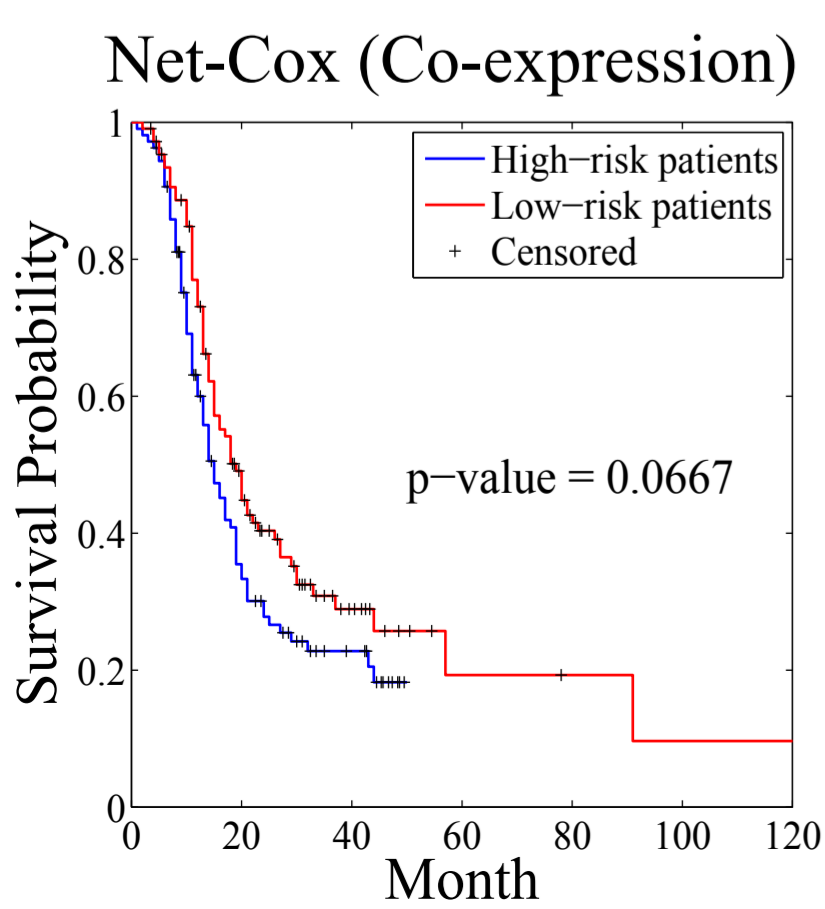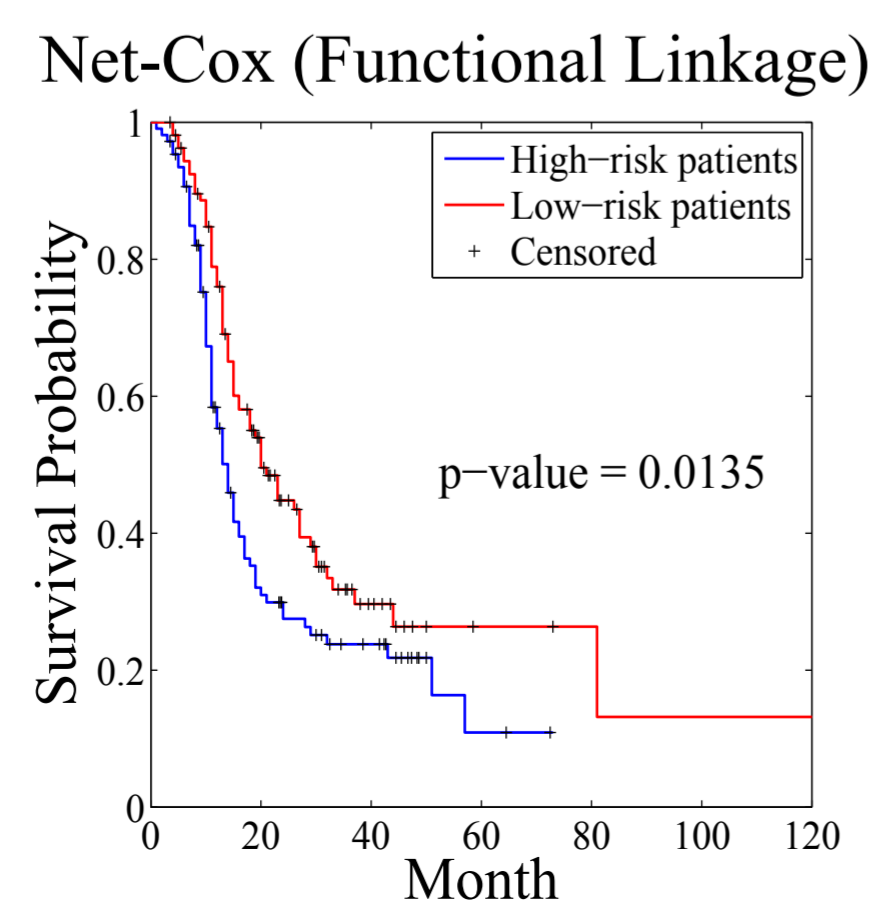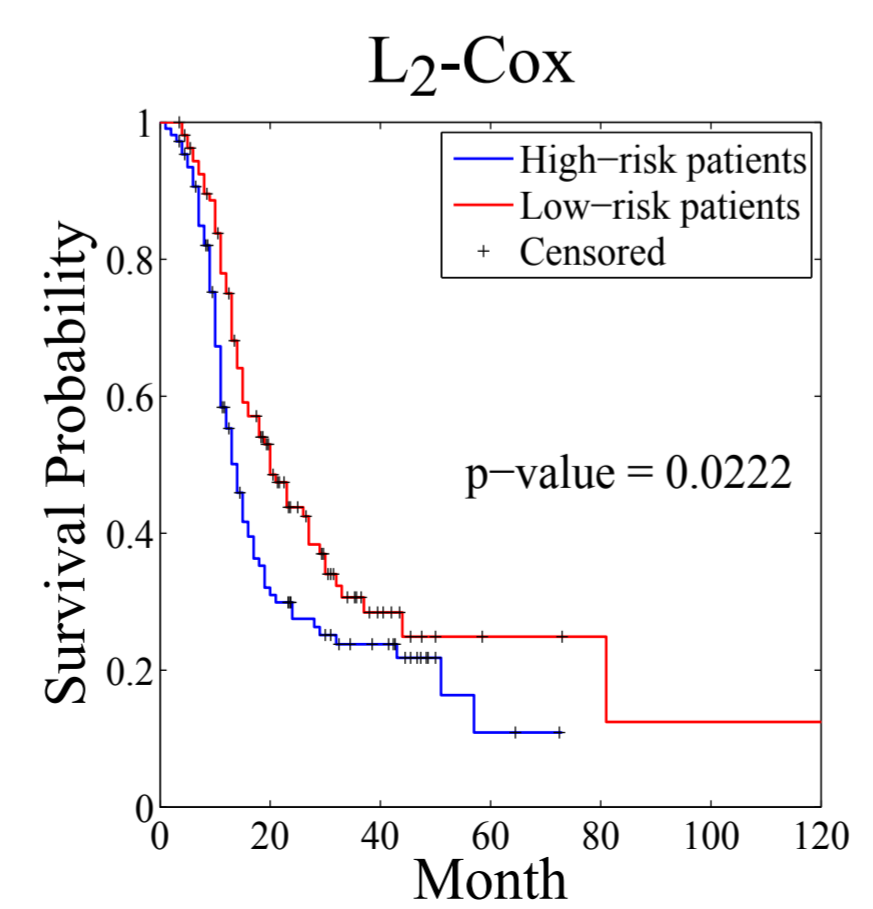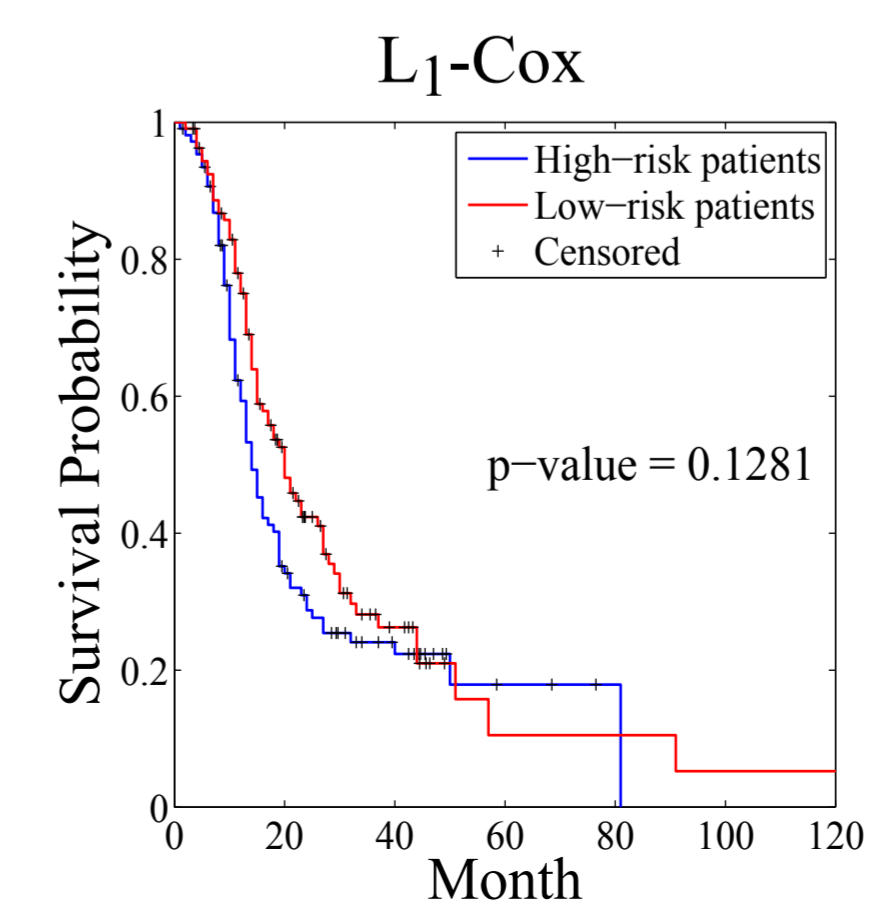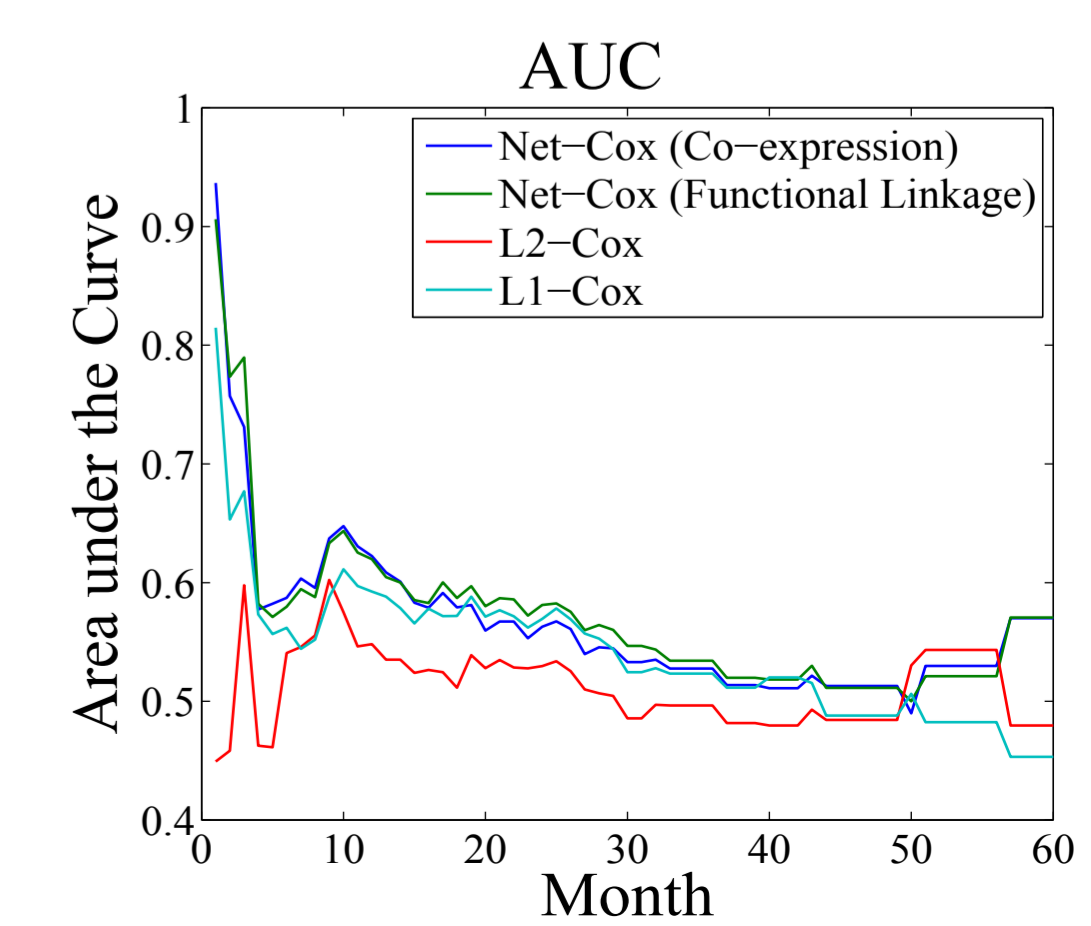

Supplement: Figure S3 — Cross-dataset survival prediction (all mappable genes). The first four columns of plots show the Kaplan-Meier survival curves for the two risk groups defined by Net-Cox (co-expression network), Net-Cox (functional linkage network), and . The fifth column of plots compare the time-dependent area under the ROC curves based on the estimated risk scores (PIs). The results are shown for the death outcome by training with TCGA dataset and test on Tothill Dataset (A), for the death outcome by training with TCGA dataset and test on Bonome Dataset (B) and for the tumor recurrence outcome by training with TCGA dataset and test on Tothill Dataset (C). (PDF) [file pcbi.1002975.s003.pdf]

**C**

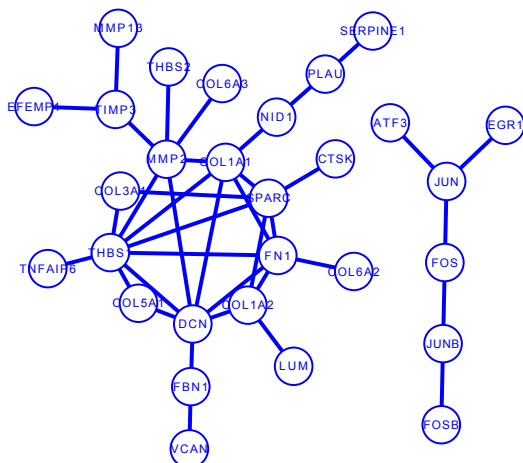

## Recurrence

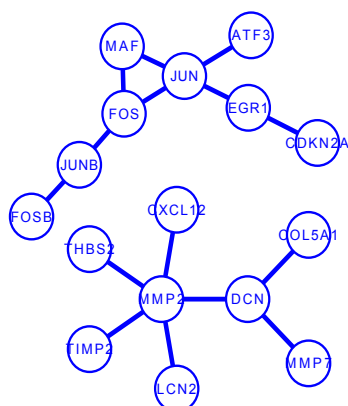

## Recurrence

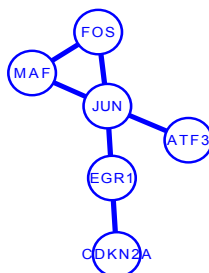

## Recurrence

Supplement: Figure S4 — Protein-Protein interaction sub-networks of marker genes identified by Net-Cox and on the TCGA dataset. (A) The PPI subnetworks identified by Net-Cox on the co-expression network. (B) The PPI subnetworks identified by Net-Cox on the functional linkage network. (C) The PPI subnetwrks identified by . (PDF) [file pcbi.1002975.s004.pdf]

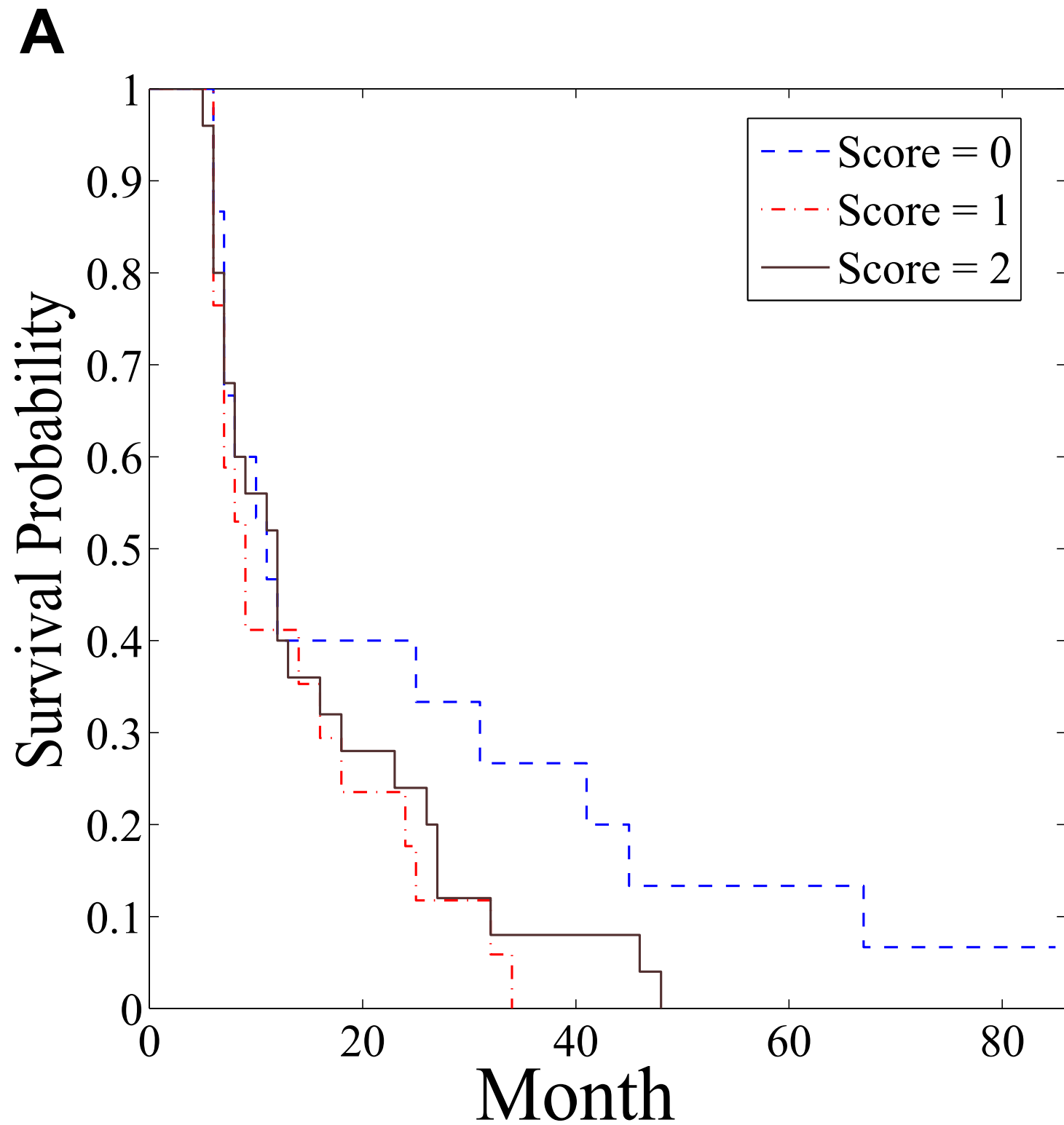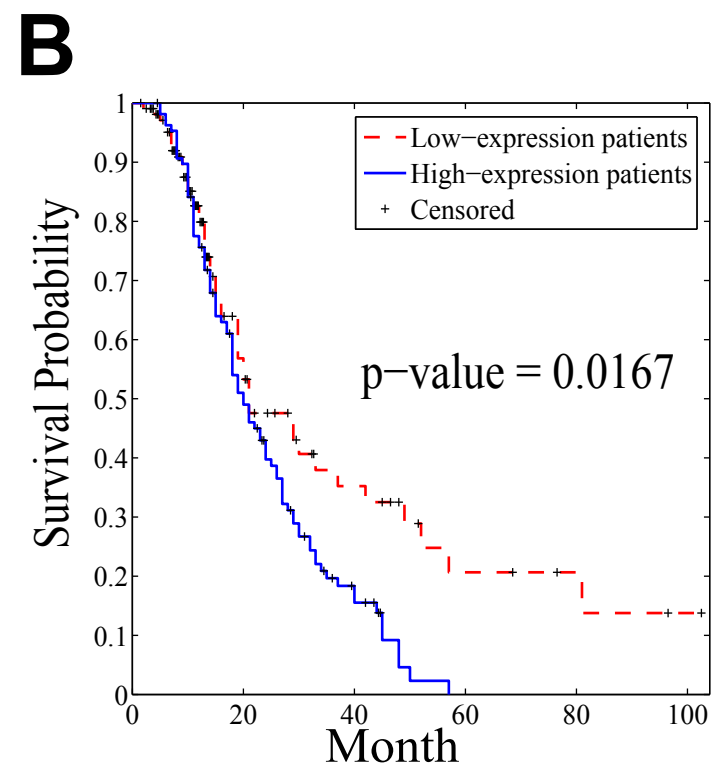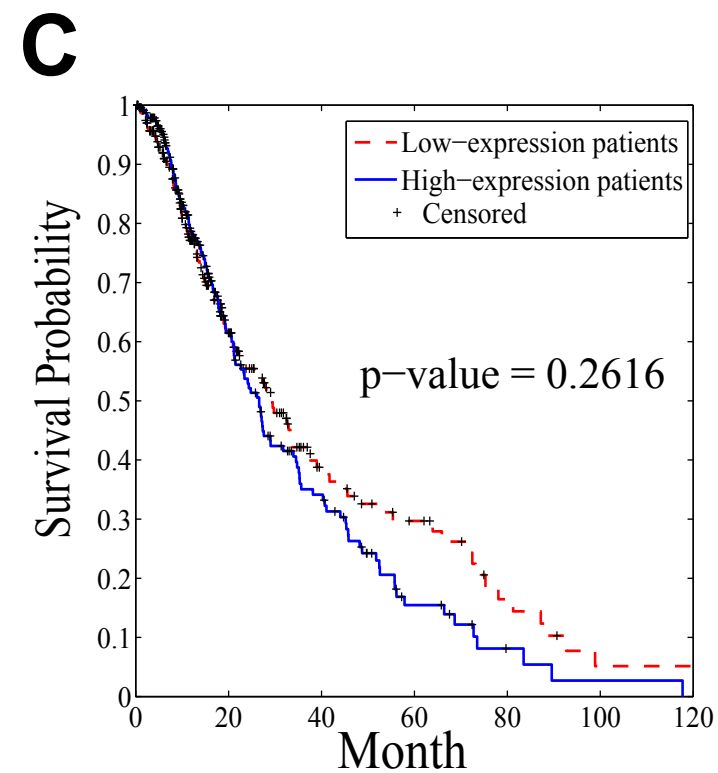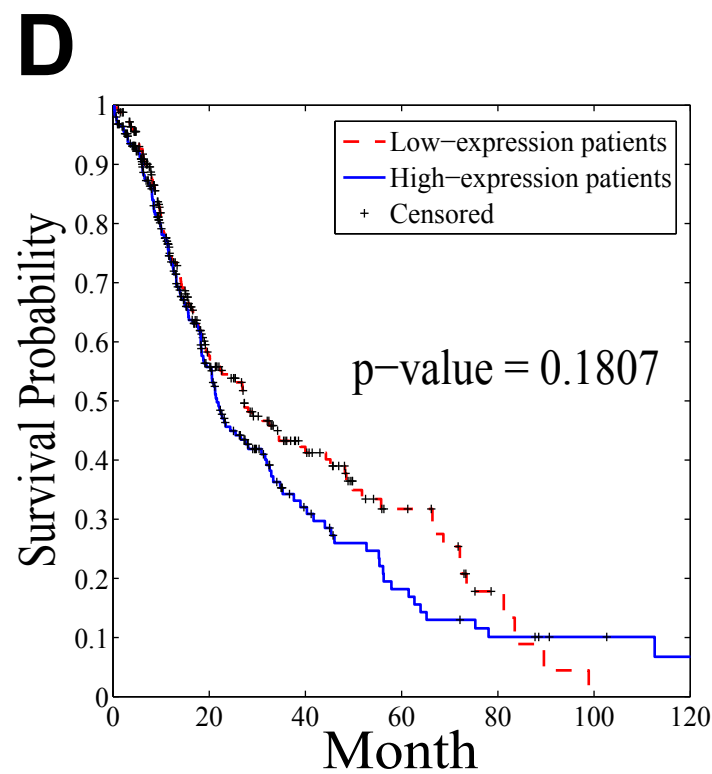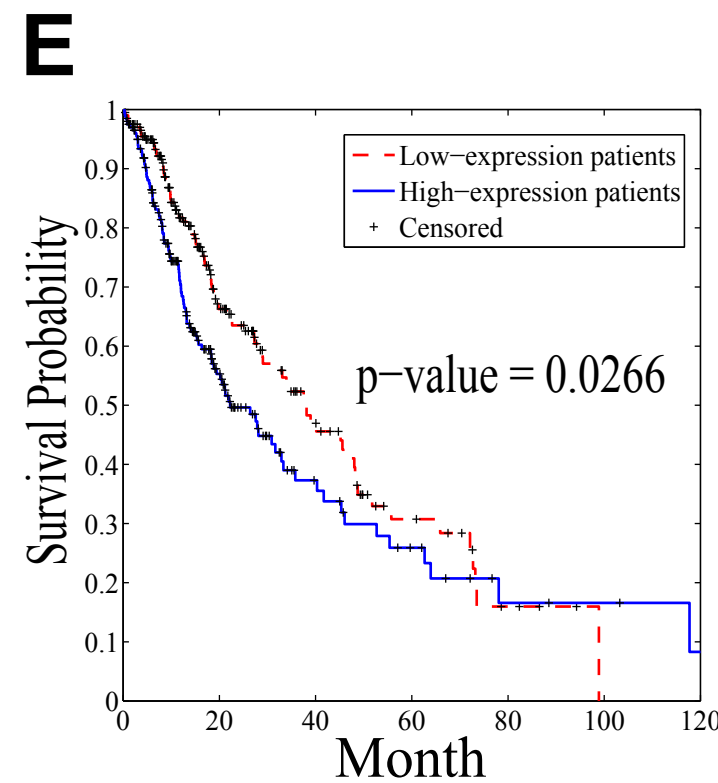

Supplement: Figure S5 — Kaplan-Meier survival plots on FBN1 expression groups. (A) Kaplan-Meier survival curve of recurrence by FBN1 staining groups. The group with low FBN1 expression has a lower recurrence rate compared with the groups with high expression after 12 month of treatment. (B) Kaplan-Meier survival curve of recurrence by the expression of FBN1 on Tothill dataset. (C)–(E) Kaplan-Meier survival curves of recurrence by the expression of FBN1 on TCGA dataset with AgilentG4502A platform, HuEx-1_0-st-v2 platform, and Affymetrix HG-U133A platform, respectively. In plots(B)–(E), the patients are divided into two groups of the same size by the expression of FBN1. (PDF) [file pcbi.1002975.s005.pdf]

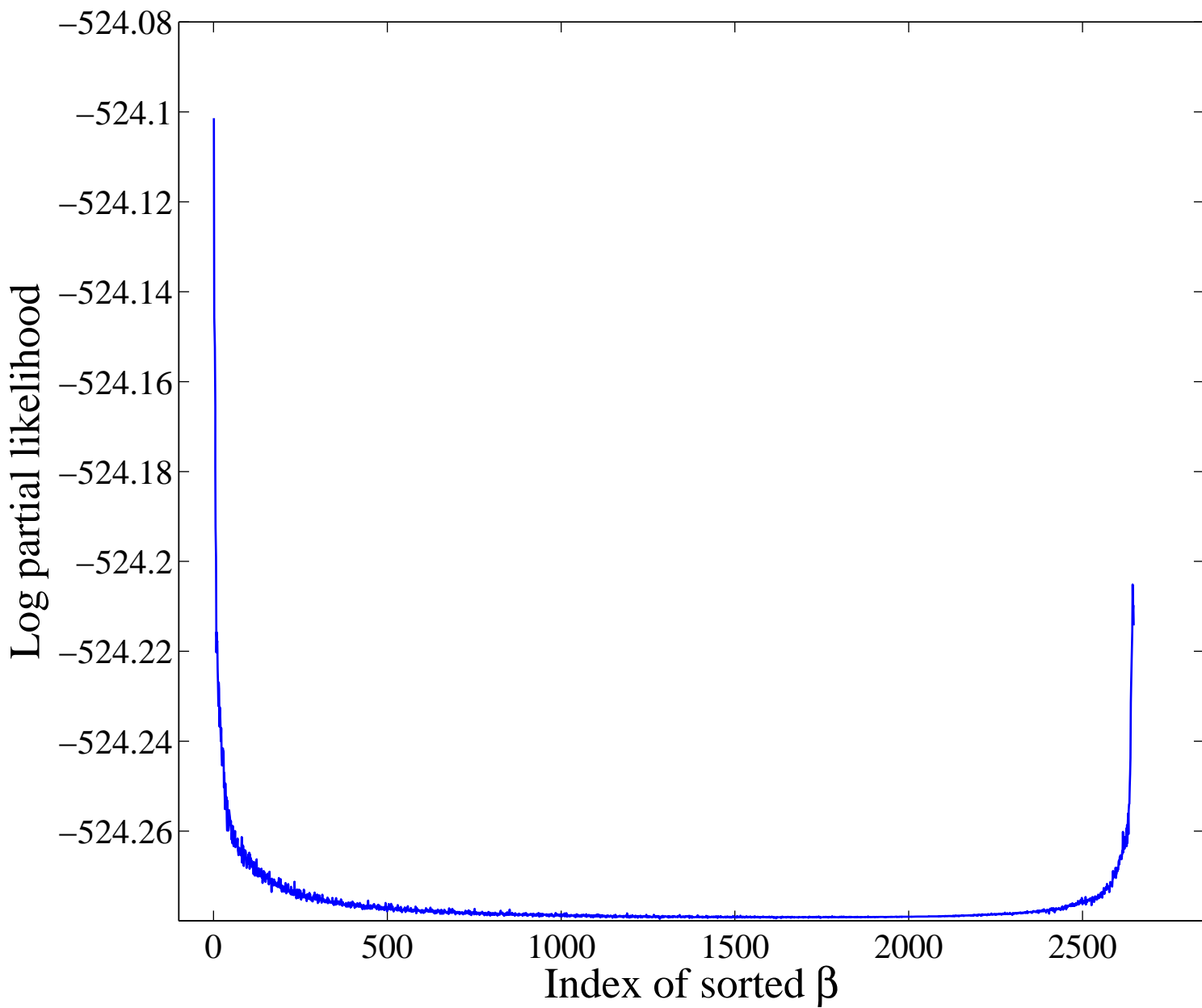

Supplement: Figure S6 — Contributions to the log-partial likelihood by each individual gene by Net-Cox on the Tothill dataset (Sloan-Kettering cancer genes). The x-axis is the index of the genes sorted by coefficients. (PDF) [file pcbi.1002975.s006.pdf]
