# Supplementary material for: Network-based Survival Analysis Reveals Subnetwork Signatures for Predicting Outcomes of Ovarian Cancer Treatment
Source: PLoS Comput Biol. 2013 Mar 21;9(3):e1002975. doi: 10.1371/journal.pcbi.1002975 (PMC3605061; doi:10.1371/journal.pcbi.1002975)
Supplement: Table S1 — Optimal parameters of Net-Cox. The parameters are selected by CVPLs in five-fold cross-validation. (a) Sloan-Kettering cancer genes. (b) All mappable genes. (PDF) [file pcbi.1002975.s007.pdf]

		Net-Cox (Co-expression)		Net-Cox (Functional Linkage)	
**Event**	**Dataset**	λ	α	λ	α
**Death**	**TCGA**	1.00E-04	0.5	1.00E-04	0.1
	**Tothill**	1.00E-04	0.1	1.00E-04	0.01
	**Bonome**	1.00E-03	0.01	1.00E-03	0.5
**Recurrence**	**TCGA**	1.00E-03	0.1	1.00E-03	0.01
	**Tothill**	1.00E-04	0.1	1.00E-04	0.5

(a) Sloan-Kettering cancer gene list

		Net-Cox (Co-expression)		Net-Cox (Functional Linkage)	
**Event**	**Dataset**	λ	α	λ	α
**Death**	**TCGA**	1.00E-04	0.5	1.00E-04	0.1
	**Tothill**	1.00E-04	0.1	1.00E-04	0.01
	**Bonome**	1.00E-03	0.1	1.00E-03	0.1
**Recurrence**	**TCGA**	1.00E-03	0.1	1.00E-03	0.1
	**Tothill**	1.00E-04	0.1	1.00E-04	0.95

(b) All the genes

Table S1
