# Supplementary material for: Network-based Survival Analysis Reveals Subnetwork Signatures for Predicting Outcomes of Ovarian Cancer Treatment
Source: PLoS Comput Biol. 2013 Mar 21;9(3):e1002975. doi: 10.1371/journal.pcbi.1002975 (PMC3605061; doi:10.1371/journal.pcbi.1002975)
Supplement: Table S2 — Statistical significance of the improvement in time-dependent AUCs in cross-dataset evaluation (Sloan-Kettering cancer genes). The R package “timeROC” (the algorithm was described in the paper “Estimating and Comparing time-dependent areas under ROC curves for censored event times with competing risks”) was used to compute the . The null hypothesis asserts that two time-dependent AUCs estimated by two models are equal. The significant smaller than 0.1 are bold. The tables show the results for the death outcome by training with TCGA dataset and test on Tothill Dataset (a), for the death outcome by training with TCGA dataset and test on Bonome Dataset (b), for the tumor recurrence outcome by training with TCGA dataset and test on Tothill Dataset (c). (PDF) [file pcbi.1002975.s008.pdf]

Quantile of the event times	10%	20%	30%	40%	50%	60%	70%	80%	90%
Event time(month)	11	16	21	24	29	33	38	44	48
Net-Cox (Co-expression) vs. L_2-Cox	0.7913	0.2312	0.5195	0.3909	0.2579	0.3719	0.6895	0.5207	0.4513
Net-Cox (Co-expression) vs. L_1-Cox	0.6195	**4.9E-05**	**4.4E-04**	**1.2E-04**	**1.7E-04**	**1.2E-04**	**0.0653**	0.2792	**0.0835**
Net-Cox (Functional Linkage) vs. L_2-Cox	0.8501	0.1186	0.2956	0.3400	0.2295	0.2875	0.6685	0.8256	0.2340
Net-Cox (Functional Linkage) vs. L_1-Cox	0.8846	**3.3E-05**	**2.4E-04**	**1.1E-04**	**1.6E-04**	**1.2E-04**	**0.0611**	0.2013	**0.0518**

(a) Test on Tothill (Death)

Quantile of the event times	10%	20%	30%	40%	50%	60%	70%	80%	90%
Event time (month)	9	15	22	28	34	43	51	65	89
Net-Cox (Co-expression) vs. L_2-Cox	0.3091	0.5509	0.5816	0.3753	**0.0635**	**0.0357**	0.1520	0.1234	**0.0230**
Net-Cox (Co-expression) vs. L_1-Cox	0.3388	0.1302	0.2294	**0.0248**	**0.0152**	**0.0749**	**0.0541**	0.1735	**0.0047**
Net-Cox (Functional Linkage) vs. L_2-Cox	0.4151	0.9300	0.5037	0.3290	**0.0408**	**0.0267**	**0.0651**	**0.0361**	**0.0345**
Net-Cox (Functional Linkage) vs. L_1-Cox	0.5911	0.2508	0.1418	**0.0120**	**0.0023**	**0.0143**	**0.0084**	**0.0265**	**6.9E-05**

(b) Test on Bonome (Death)

Quantile of the event times	10%	20%	30%	40%	50%	60%	70%	80%	90%
Event time (month)	7	9	11	13	15	19	23	30	44
Net-Cox (Co-expression) vs. L_2-Cox	0.1935	0.3518	**0.0237**	**0.0321**	**0.0590**	0.1568	0.2268	0.2838	0.8144
Net-Cox (Co-expression) vs. L_1-Cox	**0.0739**	**0.0722**	**0.0036**	**0.0448**	0.3566	0.8644	0.9035	0.7763	0.2958
Net-Cox (Functional Linkage) vs. L_2-Cox	0.1185	0.1699	**0.0087**	**0.0060**	**0.0043**	**0.0075**	**0.0251**	**0.0772**	0.6273
Net-Cox (Functional Linkage) vs. L_1-Cox	**0.0590**	**0.0280**	**0.0018**	**0.0107**	**0.0830**	0.2376	0.3500	0.7855	0.3638

(c) Test on Tothill (Recurrence)

Table S2
