# Supplementary material for: Network-based Survival Analysis Reveals Subnetwork Signatures for Predicting Outcomes of Ovarian Cancer Treatment
Source: PLoS Comput Biol. 2013 Mar 21;9(3):e1002975. doi: 10.1371/journal.pcbi.1002975 (PMC3605061; doi:10.1371/journal.pcbi.1002975)
Supplement: Table S3 — Cross validation partial likelihood (CVPL) in five-fold cross-validation (Sloan-Kettering cancer genes). (a) The death outcome of TCGA dataset. (b) The tumor recurrence outcome of TCGA dataset. (c) The death outcome of Tothill dataset. (d) The tumor recurrence outcome of Tothill dataset. (e) The death outcome of Bonome dataset. (f) . (g) Group Lasso and Sparse-Group Lasso. (PDF) [file pcbi.1002975.s009.pdf]

| | Net-Cox (Co-expression) | | | | Net-Cox (Functional Linkage) | | | | $L_2$-Cox |
|---|---|---|---|---|---|---|---|---|---|
| $\lambda \setminus \alpha$ | 0.01 | 0.1 | 0.5 | 0.95 | 0.01 | 0.1 | 0.5 | 0.95 | 1 |
| 1.00E-05 | -1864.85 | -1815.99 | -1757.76 | -1738.40 | -1743.75 | -1737.62 | -1737.21 | -1737.16 | -1737.16 |
| 1.00E-04 | -1679.82 | -1674.76 | **-1673.94** | -1674.51 | -1676.93 | **-1674.08** | -1674.48 | -1674.55 | **-1674.57** |
| 1.00E-03 | -1679.54 | -1679.82 | -1682.17 | -1683.10 | -1684.17 | -1683.17 | -1683.17 | -1683.16 | -1683.16 |
| 0.01 | -1684.64 | -1685.13 | -1685.70 | -1685.86 | -1685.83 | -1685.87 | -1685.87 | -1685.87 | -1685.87 |
| 0.1 | -1686.06 | -1686.12 | -1686.19 | -1686.20 | -1686.20 | -1686.20 | -1686.20 | -1686.20 | -1686.20 |
| 1 | -1686.22 | -1686.23 | -1686.24 | -1686.24 | -1686.24 | -1686.24 | -1686.24 | -1686.24 | -1686.24 |

**(a)** TCGA (Death)

| | Net-Cox (Co-expression) | | | | Net-Cox (Functional Linkage) | | | | $L_2$-Cox |
|---|---|---|---|---|---|---|---|---|---|
| $\lambda \setminus \alpha$ | 0.01 | 0.1 | 0.5 | 0.95 | 0.01 | 0.1 | 0.5 | 0.95 | 1 |
| 1.00E-05 | -1852.04 | -1811.50 | -1743.35 | -1720.91 | -1716.86 | -1719.15 | -1719.45 | -1719.49 | -1719.49 |
| 1.00E-04 | -1646.91 | -1643.78 | -1640.72 | -1640.31 | -1641.27 | -1640.34 | -1640.33 | -1640.31 | -1640.31 |
| 1.00E-03 | -1637.96 | **-1637.75** | -1638.81 | -1639.16 | **-1639.12** | -1639.21 | -1639.19 | -1639.19 | **-1639.19** |
| 0.01 | -1639.21 | -1639.42 | -1639.65 | -1639.71 | -1639.72 | -1639.71 | -1639.71 | -1639.71 | -1639.71 |
| 0.1 | -1639.72 | -1639.75 | -1639.77 | -1639.78 | -1639.78 | -1639.78 | -1639.78 | -1639.78 | -1639.78 |
| 1 | -1639.78 | -1639.78 | -1639.79 | -1639.79 | -1639.79 | -1639.79 | -1639.79 | -1639.79 | -1639.79 |

**(b)** TCGA (Recurrence)

| | Net-Cox (Co-expression) | | | | Net-Cox (Functional Linkage) | | | | $L_2$-Cox |
|---|---|---|---|---|---|---|---|---|---|
| $\lambda \setminus \alpha$ | 0.01 | 0.1 | 0.5 | 0.95 | 0.01 | 0.1 | 0.5 | 0.95 | 1 |
| 1.00E-05 | -688.278 | -664.544 | -636.620 | -628.431 | -628.626 | -627.972 | -627.923 | -627.915 | -627.914 |
| 1.00E-04 | -609.226 | **-607.624** | -607.875 | -608.731 | **-608.774** | -608.776 | -608.809 | -608.808 | **-608.808** |
| 1.00E-03 | -614.060 | -615.075 | -617.393 | -618.362 | -617.574 | -618.352 | -618.422 | -618.430 | -618.430 |
| 0.01 | -620.219 | -620.674 | -621.198 | -621.356 | -621.152 | -621.353 | -621.365 | -621.366 | -621.366 |
| 0.1 | -621.593 | -621.649 | -621.708 | -621.725 | -621.709 | -621.724 | -621.726 | -621.726 | -621.726 |
| 1 | -621.749 | -621.755 | -621.761 | -621.762 | -621.761 | -621.762 | -621.762 | -621.762 | -621.762 |

**(c)** Tothill (Death)

| | Net-Cox (Co-expression) | | | | Net-Cox (Functional Linkage) | | | | $L_2$-Cox |
|---|---|---|---|---|---|---|---|---|---|
| $\lambda \setminus \alpha$ | 0.01 | 0.1 | 0.5 | 0.95 | 0.01 | 0.1 | 0.5 | 0.95 | 1 |
| 1.00E-05 | -1189.16 | -1151.98 | -1100.86 | -1084.93 | -1083.45 | -1083.75 | -1083.88 | -1083.97 | -1083.98 |
| 1.00E-04 | -1034.87 | **-1032.66** | -1033.95 | -1035.84 | -1037.06 | -1036.08 | **-1035.82** | -1036.02 | **-1036.02** |
| 1.00E-03 | -1047.89 | -1048.83 | -1054.09 | -1056.52 | -1057.19 | -1056.74 | -1056.70 | -1056.70 | -1056.70 |
| 0.01 | -1061.51 | -1062.72 | -1064.45 | -1064.99 | -1064.60 | -1065.01 | -1065.02 | -1065.02 | -1065.02 |
| 0.1 | -1065.74 | -1065.93 | -1066.15 | -1066.21 | -1066.20 | -1066.21 | -1066.22 | -1066.22 | -1066.22 |
| 1 | -1066.29 | -1066.31 | -1066.33 | -1066.34 | -1066.34 | -1066.34 | -1066.34 | -1066.34 | -1066.34 |

**(d)** Tothill (Recurrence)

| | Net-Cox (Co-expression) | | | | Net-Cox (Functional Linkage) | | | | $L_2$-Cox |
|---|---|---|---|---|---|---|---|---|---|
| $\lambda \setminus \alpha$ | 0.01 | 0.1 | 0.5 | 0.95 | 0.01 | 0.1 | 0.5 | 0.95 | 1 |
| 1.00E-05 | -927.861 | -877.529 | -802.693 | -776.750 | -776.285 | -775.239 | -775.162 | -775.168 | -775.172 |
| 1.00E-04 | -661.654 | -656.089 | -649.612 | -648.112 | -649.213 | -648.141 | -648.050 | -648.039 | -648.039 |
| 1.00E-03 | **-644.371** | -644.733 | -646.189 | -647.230 | -647.418 | -647.339 | **-647.265** | -647.287 | **-647.317** |
| 0.01 | -648.411 | -649.340 | -650.544 | -650.894 | -650.847 | -650.928 | -650.919 | -650.917 | -650.917 |
| 0.1 | -651.169 | -651.329 | -651.496 | -651.537 | -651.552 | -651.541 | -651.540 | -651.540 | -651.540 |
| 1 | -651.568 | -651.585 | -651.602 | -651.606 | -651.608 | -651.607 | -651.607 | -651.607 | -651.607 |

**(e)** Bonome (Death)

| $L_1$-Cox | | |
|---|---|---|
| **Dataset (Event)** | $\lambda$ | **CVPL** |
| **TCGA (Death)** | 0.0712 | -1678.97 |
| **TCGA (Recurrence)** | 0.0972 | -1635.34 |
| **Tothill (Death)** | 0.0861 | -607.737 |
| **Tothill (Recurrence)** | 0.1308 | -1049.45 |
| **Bonome (Death)** | 0.2319 | -649.301 |

**(f)** $L_1$-Cox

| **Dataset (Event)** | **Group Lasso** | | | | **Sparse-Group Lasso ($\alpha$=0.5)** | | | |
|---|---|---|---|---|---|---|---|---|
| | **25 Clusters** | | **100 Clusters** | | **25 Clusters** | | **100 Clusters** | |
| | $\lambda$ | **CVPL** | $\lambda$ | **CVPL** | $\lambda$ | **CVPL** | $\lambda$ | **CVPL** |
| **TCGA (Death)** | 3.44E-04 | -1685.97 | 5.09E-04 | -1685.92 | 4.19E-04 | -1685.98 | 5.51E-04 | -1685.94 |
| **TCGA (Recurrence)** | 4.07E-04 | -1639.76 | 5.49E-04 | -1639.75 | 4.45E-04 | -1639.71 | 6.94E-04 | -1639.73 |
| **Tothill (Death)** | 8.36E-04 | -621.603 | 9.28E-04 | -621.502 | 8.64E-04 | -621.609 | 9.61E-04 | -621.522 |
| **Tothill (Recurrence)** | 1.42E-03 | -1066.03 | 1.74E-03 | -1066.00 | 1.71E-03 | -1066.05 | 1.74E-03 | -1066.00 |
| **Bonome (Death)** | 2.05E-03 | -651.525 | 2.05E-03 | -651.475 | 2.04E-03 | -651.516 | 2.04E-03 | -651.468 |

**(g)** Group Lasso and Sparse-Group Lasso

**Table S3**
