# Supplementary material for: Network-based Survival Analysis Reveals Subnetwork Signatures for Predicting Outcomes of Ovarian Cancer Treatment
Source: PLoS Comput Biol. 2013 Mar 21;9(3):e1002975. doi: 10.1371/journal.pcbi.1002975 (PMC3605061; doi:10.1371/journal.pcbi.1002975)
Supplement: Table S4 — Log-rank test of the test folds on five-fold cross-validation. The most significant across four models with cut-off 0.05 are bold. (a) Sloan-Kettering cancer genes and the death outcome. (b) Sloan-Kettering cancer genes and the tumor recurrence outcome. (c) All mappable genes and the death outcome. (d) All mappable genes and the tumor recurrence outcome. (PDF) [file pcbi.1002975.s010.pdf]

Dataset	Algorithm	1st fold	2nd fold	3rd fold	4th fold	5th fold
**TCGA**	**Net-Cox (Co-expression)**	1.79E-03	9.62E-01	1.63E-01	**5.17E-03**	2.35E-05
	**Net-Cox (Functional Linkage)**	9.44E-04	5.93E-01	8.41E-02	8.38E-03	**8.92E-06**
	L_2**-Cox**	**3.50E-04**	2.74E-01	2.30E-01	3.04E-01	2.12E-04
	L_1**-Cox**	3.71E-02	3.70E-01	6.82E-01	3.06E-01	4.47E-03
**Tothill**	**Net-Cox (Co-expression)**	3.99E-02	**4.49E-04**	3.89E-03	1.33E-01	1.59E-01
	**Net-Cox (Functional Linkage)**	4.30E-02	7.59E-02	**1.09E-03**	1.15E-01	1.41E-01
	L_2**-Cox**	1.25E-01	3.18E-02	3.10E-03	1.09E-01	1.24E-01
	L_1**-Cox**	**1.61E-02**	1.20E-03	6.71E-03	**2.85E-02**	2.22E-01
**Bonome**	**Net-Cox (Co-expression)**	3.24E-01	**2.91E-02**	**7.17E-03**	7.56E-01	7.49E-01
	**Net-Cox (Functional Linkage)**	4.03E-01	3.67E-02	2.27E-02	2.87E-01	9.51E-01
	L_2**-Cox**	1.61E-01	8.53E-02	4.69E-02	8.30E-01	6.74E-01
	L_1**-Cox**	5.94E-01	2.81E-01	4.16E-01	3.89E-01	3.21E-01

(a) Sloan-Kettering Cancer Gene List (Death)

Dataset	Algorithm	1st fold	2nd fold	3rd fold	4th fold	5th fold
**TCGA**	**Net-Cox (Co-expression)**	4.39E-01	3.44E-01	6.14E-01	1.64E-01	**1.82E-02**
	**Net-Cox (Functional Linkage)**	7.55E-01	4.84E-01	9.39E-01	4.28E-01	9.50E-02
	L_2**-Cox**	7.63E-01	4.29E-01	4.47E-01	1.84E-01	2.34E-01
	L_1**-Cox**	1.01E-01	2.88E-01	5.89E-01	4.09E-01	1.99E-02
**Tothill**	**Net-Cox (Co-expression)**	5.78E-05	4.75E-01	8.24E-03	**8.86E-06**	**3.88E-05**
	**Net-Cox (Functional Linkage)**	6.20E-05	5.06E-01	**2.40E-03**	8.85E-06	1.67E-04
	L_2**-Cox**	**2.30E-05**	3.00E-01	6.60E-02	6.98E-04	1.33E-03
	L_1**-Cox**	5.68E-03	5.74E-01	1.67E-02	2.11E-01	1.59E-02

(b) Sloan-Kettering Cancer Gene List (Recurrence)

Dataset	Algorithm	1st fold	2nd fold	3rd fold	4th fold	5th fold
**TCGA**	**Net-Cox (Co-expression)**	1.44E-03	3.66E-01	9.91E-02	4.64E-03	**4.49E-05**
	**Net-Cox (Functional Linkage)**	**1.11E-03**	3.11E-01	5.15E-02	**4.48E-03**	7.09E-05
	L_2**-Cox**	5.02E-03	3.88E-01	2.74E-01	1.54E-01	5.18E-04
	L_1**-Cox**	4.54E-02	5.77E-02	9.37E-02	9.79E-01	1.26E-02
**Tothill**	**Net-Cox (Co-expression)**	**1.30E-02**	**2.22E-03**	**8.06E-04**	2.62E-02	1.10E-01
	**Net-Cox (Functional Linkage)**	2.48E-02	2.28E-03	**8.06E-04**	2.62E-02	1.10E-01
	L_2**-Cox**	3.86E-02	5.60E-03	1.06E-03	1.86E-02	2.94E-01
	L_1**-Cox**	7.82E-02	2.59E-02	1.15E-02	**1.73E-02**	1.52E-01
**Bonome**	**Net-Cox (Co-expression)**	6.14E-01	**2.12E-02**	**3.30E-03**	8.79E-01	1.94E-01
	**Net-Cox (Functional Linkage)**	7.02E-01	2.13E-02	6.18E-03	8.93E-01	1.94E-01
	L_2**-Cox**	5.69E-01	4.63E-02	8.85E-03	3.62E-01	4.44E-01
	L_1**-Cox**	4.90E-01	6.84E-02	2.93E-01	2.47E-01	6.42E-02

(c) All the genes (Death)

Dataset	Algorithm	1st fold	2nd fold	3rd fold	4th fold	5th fold
**TCGA**	**Net-Cox (Co-expression)**	1.56E-01	2.35E-01	1.91E-01	1.29E-01	**2.57E-02**
	**Net-Cox (Functional Linkage)**	9.68E-01	1.71E-01	5.38E-01	3.22E-01	9.79E-02
	L_2**-Cox**	7.52E-01	9.42E-02	3.16E-01	1.98E-01	1.82E-01
	L_1**-Cox**	2.23E-01	7.37E-02	7.41E-01	6.33E-01	3.22E-02
**Tothill**	**Net-Cox (Co-expression)**	**1.77E-05**	1.97E-01	**1.03E-03**	**3.05E-04**	**2.96E-06**
	**Net-Cox (Functional Linkage)**	2.75E-05	1.62E-01	5.36E-02	8.21E-04	9.69E-05
	L_2**-Cox**	2.75E-05	1.62E-01	5.14E-02	8.21E-04	1.48E-03
	L_1**-Cox**	8.11E-04	1.03E-01	3.15E-01	1.31E-02	2.15E-02

(d) All the genes (Recurrence)

Table S4
