# Supplementary material for: Network-based Survival Analysis Reveals Subnetwork Signatures for Predicting Outcomes of Ovarian Cancer Treatment
Source: PLoS Comput Biol. 2013 Mar 21;9(3):e1002975. doi: 10.1371/journal.pcbi.1002975 (PMC3605061; doi:10.1371/journal.pcbi.1002975)
Supplement: Table S5 — Log-rank test in cross-dataset evaluation (all mappable genes). The survival prediction performance on Tothill and Bonome datasets using the Cox models trained with TCGA dataset are reported. (PDF) [file pcbi.1002975.s011.pdf]

| | Test Dataset | Net-Cox (Co-exp) | Net-Cox (FL) | $L_2$−Cox | $L_1$−Cox |
|---|---|---|---|---|---|
| **Death** | **Tothill** | 7.3516E-06 | 7.7815E-07 | 1.8205E-05 | 0.0016 |
| | **Bonome** | 3.2107E-06 | 5.3008E-06 | 9.5390E-06 | 0.0017 |
| **Recurrence** | **Tothill** | 0.0667 | 0.0135 | 0.0222 | 0.1281 |

**Table S5**
