# Supplementary material for: Network-based Survival Analysis Reveals Subnetwork Signatures for Predicting Outcomes of Ovarian Cancer Treatment
Source: PLoS Comput Biol. 2013 Mar 21;9(3):e1002975. doi: 10.1371/journal.pcbi.1002975 (PMC3605061; doi:10.1371/journal.pcbi.1002975)
Supplement: Table S6 — Enriched GO terms by the signature genes of death outcome. The in scale are shown for the enriched GO terms. (PDF) [file pcbi.1002975.s012.pdf]

Death			
**GO terms**	**Net-Cox Co-expression**	**Net-Cox Functional Linkage**	L_2**-Cox**
GO:0031012:extracellular matrix	18.944	22.990	21.794
GO:0005578:proteinaceous extracellular matrix	18.783	22.867	21.652
GO:0044421:extracellular region part	13.091	22.059	21.186
GO:0005576:extracellular region	8.823	18.087	17.455
GO:0044420:extracellular matrix part	13.841	12.713	11.351
GO:0032502:developmental process	9.323	11.432	9.843
GO:0048731:system development	8.684	11.361	9.777
GO:0007275:multicellular organismal development	8.122	11.289	9.662
GO:0048856:anatomical structure development	9.489	11.284	9.270
GO:0032501:multicellular organismal process	6.817	11.261	10.235
GO:0005201:extracellular matrix structural constituent	7.576	10.405	8.857
GO:0001501:skeletal system development	8.189	9.398	8.349
GO:0005581:collagen	9.054	5.899	4.428
GO:0005583:fibrillar collagen	8.976	4.996	3.209
GO:0030198:extracellular matrix organization	8.776	4.561	3.466
GO:0048513:organ development	7.671	8.682	7.989
GO:0009653:anatomical structure morphogenesis	3.734	8.170	7.438
GO:0005515:protein binding	7.841	4.584	4.315

Table S6
