# Supplementary material for: Network-based Survival Analysis Reveals Subnetwork Signatures for Predicting Outcomes of Ovarian Cancer Treatment
Source: PLoS Comput Biol. 2013 Mar 21;9(3):e1002975. doi: 10.1371/journal.pcbi.1002975 (PMC3605061; doi:10.1371/journal.pcbi.1002975)
Supplement: Table S7 — Enriched GO terms by the signature genes of recurrence. The in scale are shown for the enriched GO terms. A “X” denotes a larger than . (PDF) [file pcbi.1002975.s013.pdf]

Recurrence			
**GO terms**	**Net-Cox Co-expression**	**Net-Cox Functional Linkage**	L_2**-Cox**
GO:0044421:extracellular region part	18.005	12.611	10.146
GO:0031012:extracellular matrix	17.972	9.071	6.075
GO:0005578:proteinaceous extracellular matrix	17.965	9.464	5.674
GO:0005576:extracellular region	13.401	16.120	12.989
GO:0032502:developmental process	14.785	9.387	8.740
GO:0048856:anatomical structure development	14.514	9.996	10.008
GO:0048731:system development	14.251	11.061	10.330
GO:0007275:multicellular organismal development	13.553	10.139	8.788
GO:0009605:response to external stimulus	13.458	8.044	7.280
GO:0044420:extracellular matrix part	12.777	4.895	2.740
GO:0048513:organ development	11.145	8.228	7.559
GO:0005581:collagen	11.045	4.371	X
GO:0032501:multicellular organismal process	9.823	10.101	8.774
GO:0005583:fibrillar collagen	9.137	3.082	X
GO:0030198:extracellular matrix organization	8.996	3.510	X
GO:0001501:skeletal system development	8.165	4.134	2.662
GO:0009653:anatomical structure morphogenesis	8.163	3.030	2.139
GO:0042221:response to chemical stimulus	8.160	2.273	X
GO:0005615:extracellular space	7.434	5.447	4.774
GO:0005201:extracellular matrix structural constituent	7.391	3.084	X
GO:0005515:protein binding	7.114	5.843	6.217
GO:0030199:collagen fibril organization	7.044	2.756	X

Table S7
